# Dual Recognition of Sialic Acid and αGal Epitopes by the VP8* Domains of the Bovine Rotavirus G6P[5] WC3 and of Its Mono-reassortant G4P[5] RotaTeq Vaccine Strains

**DOI:** 10.1128/JVI.00941-19

**Published:** 2019-08-28

**Authors:** Mia Madel Alfajaro, Ji-Yun Kim, Laure Barbé, Eun-Hyo Cho, Jun-Gyu Park, Mahmoud Soliman, Yeong-Bin Baek, Mun-Il Kang, Soo Hyun Kim, Geun-Joong Kim, Sang-Ik Park, Jacques Le Pendu, Kyoung-Oh Cho

**Affiliations:** a Laboratory of Veterinary Pathology, College of Veterinary Medicine, Chonnam National University, Gwangju, Republic of Korea; b CRCINA, Inserm, Université d’Angers, Université de Nantes, Nantes, France; c Laboratory of Medicine, Chonnam National University Hwasun Hospital, Hwasun, Republic of Korea; d Department of Biological Sciences, College of Natural Sciences, Chonnam National University, Gwangju, Republic of Korea; Instituto de Biotecnologia/UNAM

**Keywords:** αGal, ligands, rotavirus, sialic acid

## Abstract

Group A rotaviruses, an important cause of severe diarrhea in children and young animals, initiate infection via interactions of the VP8* domain of the VP4 spike protein with cell surface sialic acids (SAs) or histo-blood group antigens (HBGAs). Although the bovine G6P[5] WC3 strain is an important animal pathogen and is also used in the bovine-human reassortant RotaTeq vaccine, the receptor(s) for the VP8* domain of WC3 and its reassortant strains have not yet been identified. In the present study, HBGA- and saliva-binding assays showed that both G6P[5] WC3 and mono-reassortant G4P[5] strains recognized the αGal HBGA. The infectivity of both P[5]-bearing strains was significantly reduced in αGal-free MA-104 cells by pretreatment with a broadly specific neuraminidase or by coincubation with the α2,6-linked SA-specific Sambucus nigra lectin, but not by the α2,3-linked specific sialidase or by Maackia amurensis lectin. Free NeuAc and the αGal trisaccharide also prevented the infectivity of both strains. This indicated that both P[5]-bearing strains utilize α2,6-linked SA as a ligand on MA104 cells. However, the two strains replicated in differentiated bovine small intestinal enteroids and in their human counterparts that lack α2,6-linked SA or αGal HBGA, suggesting that additional or alternative receptors such as integrins, hsp70, and tight-junction proteins bound directly to the VP5* domain can be used by the P[5]-bearing strains to initiate the infection of human cells. In addition, these data also suggested that P[5]-bearing strains have potential for cross-species transmission.

**IMPORTANCE** Group A rotaviruses initiate infection through the binding of the VP8* domain of the VP4 protein to sialic acids (SAs) or histo-blood group antigens (HBGAs). Although the bovine G6P[5] WC3 strain is an important animal pathogen and is used as the backbone in the bovine-human reassortant RotaTeq vaccine, the receptor(s) for their P[5] VP8* domain has remained elusive. Using a variety of approaches, we demonstrated that the WC3 and bovine-human mono-reassortant G4P[5] vaccine strains recognize both α2,6-linked SA and αGal HBGA as ligands. Neither ligand is expressed on human small intestinal epithelial cells, explaining the absence of natural human infection by P[5]-bearing strains. However, we observed that the P[5]-bearing WC3 and G4P[5] RotaTeq vaccine strains could still infect human intestinal epithelial cells. Thus, the four P[5] RotaTeq vaccine strains potentially binding to additional alternative receptors may be efficient and effective in providing protection against severe rotavirus disease in human.

## Abstract (precis)

Group A rotaviruses initiate infection through the binding of the VP8* domain of the VP4 protein to sialic acids (SAs) or histo-blood group antigens (HBGAs). Although the bovine G6P[5] WC3 strain is an important animal pathogen and is used as the backbone in the bovine-human reassortant RotaTeq vaccine, the receptor(s) for their P[5] VP8* domain has remained elusive. Using a variety of approaches, we demonstrated that the WC3 and bovine-human mono-reassortant G4P[5] vaccine strains recognize both α2,6-linked SA and αGal HBGA as ligands. Neither ligand is expressed on human small intestinal epithelial cells, explaining the absence of natural human infection by P[5]-bearing strains. However, we observed that the P[5]-bearing WC3 and G4P[5] RotaTeq vaccine strains could still infect human intestinal epithelial cells. Thus, the four P[5] RotaTeq vaccine strains potentially binding to additional alternative receptors may be efficient and effective in providing protection against severe rotavirus disease in human.

## INTRODUCTION

Diarrhea is the second largest cause of mortality worldwide among all infectious disorders in children younger than 5 years (1). Group A rotaviruses (RVAs), members of the genus *Rotavirus* within the family *Reoviridae*, are the single most important cause of severe diarrheal illness in infants and young children in both developed and developing countries worldwide (2, 3). In humans, RVAs are estimated to cause 200,000 deaths per year in children under the age of 5 years, mostly in developing countries (4). RVAs are also important veterinary pathogens in a wide variety of young animals, including cattle, pigs, sheep, and poultry (3). The mature RVA virion, a triple-layered particle, contains 11 segments of the double-stranded RNA genomes which encode six structural (VP1 to VP4, VP6, and VP7) and six nonstructural (NSP1 to NSP6) proteins (2). The unique segmented nature of RVA genomes can result in reassortment events between different viruses during coinfection of a single host and can induce the formation of progeny viruses with novel or atypical phenotypes.

The outermost layer of the RVA particles consists of two proteins, VP4 and VP7. The VP4 protein is cleaved into VP8* and VP5* by a protease, both of which are essential for the virus to host-cell entry (5–7). The VP7 protein functions at the postattachment step, interacting with integrins and heat shock cognate proteins on the cell surface (5, 7). The VP4 and VP7 proteins independently elicit neutralizing antibodies, which induce protective immunity; these proteins are used to classify the RVA strains into G (for glycoprotein, VP7) and P (for protease-sensitive, VP4) serotypes and genotypes (2, 6). To date, 35 G and 50 P genotypes have been ratified by the Rotavirus Classification Working Group (https://rega.kuleuven.be/cev/viralmetagenomics/virus-classification/rcwg), with various combinations of G and P genotypes identified in humans and animals from different countries (8–12).

RVAs enter cells using a complex multistep process, in which the first interaction of the virus with the cell surface carbohydrate moieties is mediated by the VP8* domain of the outer capsid protein VP4 (5, 7). The VP8* domain of some animal RVAs has been shown to require *N*-acetylneuraminic acid (also termed sialic acid [SA]), found in complex glycans on cell membrane glycoproteins or glycolipids, as demonstrated by dramatically reduced infectivity following neuraminidase (NA) pretreatment of cells (5, 7, 13). These strains have consequently been classified as NA sensitive (5, 7, 13). In contrast, the infectivity of many animal and most human RVA strains is much less affected by NA pretreatment of cells; these have consequently been classified as NA insensitive (5, 7, 14, 15). Internal SAs within oligosaccharide structures are less sensitive or even insensitive to NA, and therefore some NA-insensitive strains have been shown to bind to the internal SAs of glycolipids, such as the GM1a ganglioside (16, 17).

Human histo-blood group antigens (HBGAs), found on the surface of epithelial cells and in mucosal secretions, are binding partners of VP8* domains of a number of NA-insensitive human RVA strains (18–22). Based on their VP8* sequences, RVAs have been assigned to five P genogroups (P[I] to P[V]), of which viruses in P[I], P[IV], and P[V] genogroups mainly infect animals, P[II] genogroup viruses infect humans, and the P[III] genogroup viruses infect both animals and humans (21). Some SA-sensitive animals strains (P[1], P[2], P[3], and P[7]) form a subcluster within P[I], while all three major P genotypes of human RVAs (P[4], P[6], and P[8]) are clustered in the P[II] genogroup (21) and have been found to interact with type 1 precursor, H type 1, or Lewis^b^ HBGAs (18, 21, 23). Interestingly, P[9], P[14], and P[25] genotypes within the P[III] genogroup, which are rarely found in human infections, recognize A type HBGA (18, 20, 21) and also bind to the A antigens of porcine and bovine mucins, suggesting that the A antigen may mediate cross-species RVA transmission (21). These findings indicate that the interaction between P genotypes and cell surface carbohydrate moieties (terminal or internal SAs and HBGAs) shows strain specificity that appears to be genetically related (18, 20, 21). In addition, the naturally occurring bovine-human neonatal reassortant RVA strain, G10P[11], belongs to the P[IV] genogroup and binds to the H type-2 precursor HBGA and repeated lactosamine sequences, which are expressed in young humans and animals (24), thus supporting the potential for zoonotic RVA transmission (21).

Two human vaccines, Rotarix and RotaTeq, have been licensed and are used for the prevention of RVAs worldwide (2, 25). Rotarix is a monovalent live-attenuated vaccine generated by multiple tissue culture passages of human RVA G1P[8] strain 89-12. The RotaTeq vaccine contains five human-bovine reassortant RVAs, consisting of a bovine (WC3) backbone, with human RVA surface proteins representative of the most common G (G1 to G4) or P (P1A[8]) genotypes worldwide (2, 25). Although the WC3 strain is reported to be NA insensitive (15) and belongs to the P[1] genogroup (21), it is unknown whether its VP8* domain binds to SAs or HBGAs. The objective of this study was therefore to determine which carbohydrate moieties could be recognized by the VP8* domains of both the bovine G6P[5] WC3 strain and the human-bovine reassortant G4P[5] strain. We found that these two P[5]-bearing strains utilized both α2,6-linked SAs and αGal HBGA as ligands. Moreover, human intestinal enteroids (HIEs), which express neither α2,6-linked SAs nor αGal HBGA, allowed the replication of both bovine G6P[5] WC3 and human-bovine mono-reassortant G4P[5] strains. This suggests that the four P[5]-bearing strains in the RotaTeq vaccine may be able to initiate infection in humans through other receptors, while the bovine G6P[5] WC3 strain has the potential for zoonotic transmission.

## RESULTS

### HBGA binding assay showed that P[5]-bearing strains use the αGal HBGA as a ligand.

**FIG 1:**
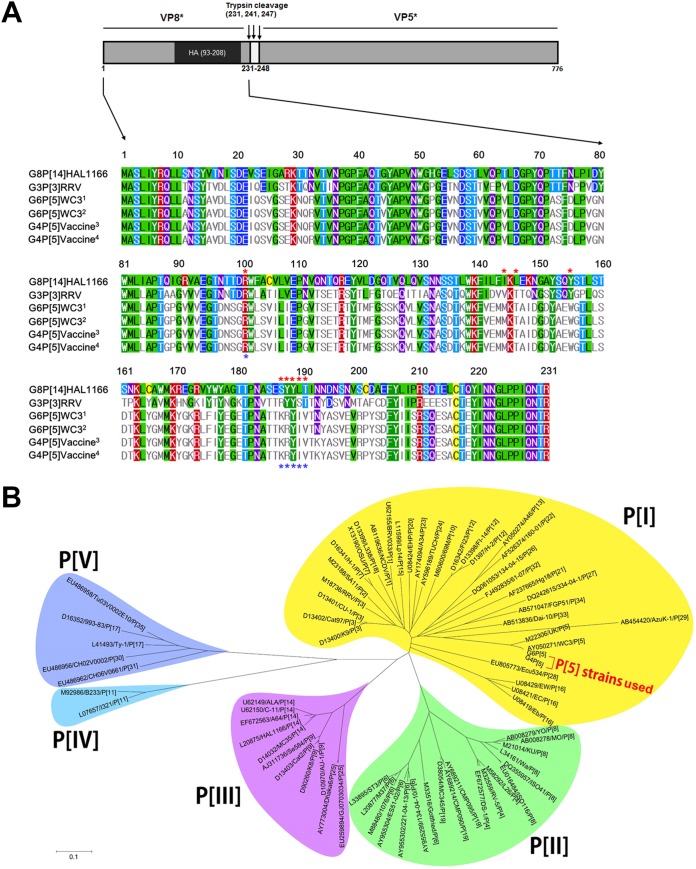
Full-length VP8* amino acid sequence and phylogenetic analyses. (A) Structural features of the VP8* domain of the spike protein VP4 shows the VP8* (left) and VP5* (right) domains with trypsin cleavage sites. The VP8* domains of the original GenBank-deposited WC3 (G6P[5] WC3 marked by a superscript 1) and RotaTeq (G4P[5] RotaTeq marked by a superscript 3) strains, and G6P[5] (marked by a superscript 2) and G4P[5] RotaTeq (marked by a superscript 4) strains used in this study, and control G3P[3] RRV and G8P[14] HAL1166 strains were 231 amino acids in length. The VP8* hemagglutination (HA) domain (amino acids 93 to 208) is shown in the schematic diagram. In the multiple sequence alignment, the amino acids at the sialic acid (SA) binding site of P[I] strains (see panel B) are marked with red asterisks (amino acids 101, 144, 146, 155, and 187 to 190). Amino acids at the blood group A binding site of P[III] strains are marked with blue asterisks (amino acids 101 and 187 to 190). The information regarding the SA and HBGA binding sites is from Isa et al. (26) and Hu et al. (27). The full-length VP8* amino acid sequences of the bovine G6P[5] WC3 strain and bovine-human mono-reassortant G4P[5] strain used in this study show only one amino acid substitution at residue 193 (N193K). (B) Phylogenetic analysis of the full-length VP8* amino acid sequence was performed using the neighbor-joining method. Note that the VP8* domain of both strains used in this study belongs to the P[I] genogroup, which is closely related to the GenBank-registered WC3 strain. Sequence information for the 71 selected strains is shown in the order of GenBank accession number, strain name, and P genotype. Calibration bar indicates the nucleotide substitutions per site.

A bovine-human mono-reassortant G4P[5] strain, carrying a human G4 gene in the genetic background of the bovine WC3 RVA strain, is currently used as one component of the pentavalent RVA vaccine, RotaTeq (25). Prior to identifying their ligand(s), the VP8* domains from the current G6P[5] WC3 strain and G4P[5] RotaTeq vaccine strain being used as one component of RotaTeq vaccine were amplified and sequenced, and then their nucleotide and deduced amino acid sequences were compared. As shown in Fig. 1A, the sequences from both strains used in this study were 696 nucleotides long, encoding 231 amino acid residues. We then compared the nucleotide and deduced amino acid sequences of the VP8* domain from the WC3 and RotaTeq strains with those of the original WC3 and G4P[5] RotaTeq strains deposited in the GenBank database. The results showed that 31 nucleotide substitutions were detected between the current WC3 and original GenBank database-deposited WC3 strains without any amino acid substitution (Fig. 1A). Compared to the original GenBank-deposited RotaTeq vaccine strain, the current G4P[5] RotaTeq vaccine strain had five nucleotide substitutions without causing amino acid substitutions (Fig. 1A). However, there was one amino acid substitution between G6P[5] WC3 and mono-reassortant RotaTeq G4P[5] strains at amino acid 193 (N193K) (Fig. 1A). This amino acid substitution was within the hemagglutination domain, but outside the known receptor-binding sites of P[I], P[II], P[III], and P[IV] (19, 26, 27), indicating that VP8* domains of both strains use the same receptor. Phylogenetic analysis of the deduced amino acid sequences also showed that both strains were closely related to each other and belonged to the P[I] genogroup, as reported previously (Fig. 1B) (21).

**FIG 2:**
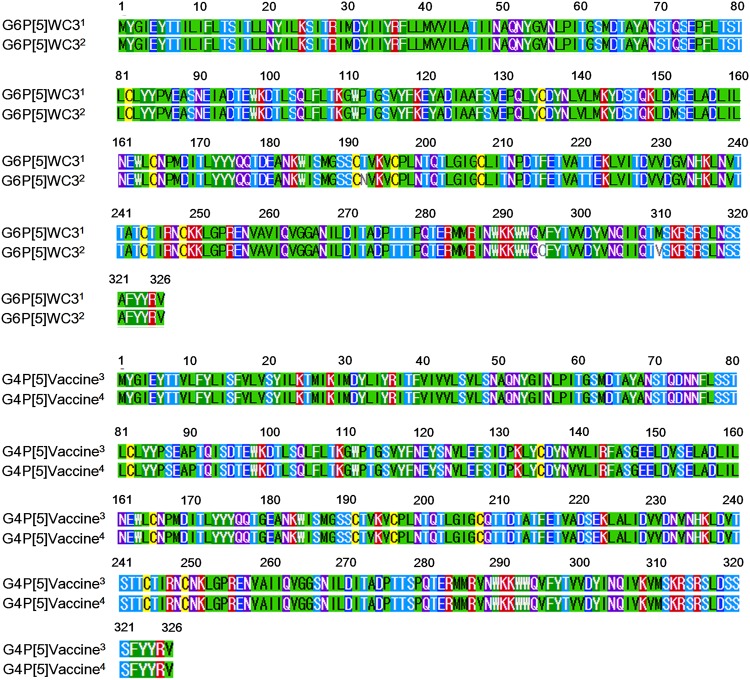
Full-length VP7 amino acid sequence. The VP7 amino acids of the original GenBank-deposited WC3 (G6P[5] WC3 marked by a superscript 1) and RotaTeq (G4P[5] RotaTeq marked by a superscript 3) strains, and G6P[5] (marked by a superscript 2) and G4P[5] RotaTeq (marked by a superscript 4) strains used in this study were 326 amino acids in length. Comparison of deduced amino acid sequences of VP7 gene between the current WC3 and original GenBank database-deposited WC3 strains showed that three amino acid substitutions were found at amino acids 192 (N192T), 295 (C296V), and 310 (V310M). The full-length VP7 amino acid sequences of both current and original GenBank database-deposited G4P[5] strains were identical.

To examine whether amino acid changes of VP7 proteins between the current G6P[5] WC3 and GenBank database-deposited WC3 strains and current G4P[5] and GenBank database-deposited G4P[5] strains may influence the NA sensitivity of VP8* domain, their coding regions were amplified and sequenced, and then their nucleotide and deduced amino acid sequences were compared to those of GenBank database-deposited strains. Both strains used in this study were 1,062 nucleotides in length, encoding 326 amino acid residues, which were identical to those of their original GenBank database-deposited strains (Fig. 2). Comparison of nucleotide and deduced amino acid sequences of VP7 gene between the current WC3 and original GenBank database-deposited WC3 strains showed that 15 nucleotide substitutions were detected, resulting in three amino acid substitutions at amino acids 192 (N192T), 295 (C296V), and 310 (V310M) (Fig. 2). In addition, even four nucleotide substitutions in VP7 genes were observed between the current and GenBank database-deposited G4P[5] RotaTeq vaccine strains, they were all silent mutations (Fig. 2).

**FIG 3:**
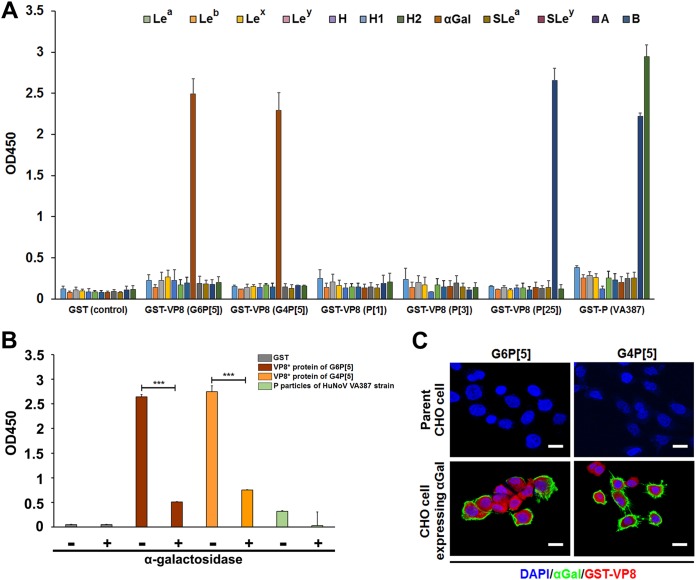
Binding of the recombinant VP8* domain of bovine G6P[5] WC3 and bovine-human mono-reassortant G4P[5] strains to synthetic histo-blood group antigens (HBGAs) and CHO cells after GGTA1 transfection. (A) The binding abilities of the recombinant GST- VP8* domains corresponding to P[5], P[1], P[3], and P[25] RVA strains and the GST-P domain of human norovirus strain VA387 (GII.4) were determined using horseradish peroxidase (HRP)-conjugated streptavidin. HBGA binding was visualized using TMB, which was measured at 450 nm in three independent experiments. Error bars represent means ± the standard deviations (SD). (B) The αGal epitope was removed from the synthetic Galα3Galβ4GlcNAcβ HBGA using α-galactosidase. The binding specificity to the VP8* domain of the bovine G6P[5] WC3 and bovine-human mono-reassortant G4P[5] strains and to P particles of the human norovirus VA387 strain was determined using an HBGA binding assay. (C) Mock-transfected parent CHO cells or GGTA1-transfected CHO cells were coincubated with FITC-conjugated anti-αGal MAb and AF594-labeled VP8* proteins of either G6P[5] WC3 or G4P[5] RotaTeq vaccine strains. No labeling was visible on mock-transfected CHO cells that lack αGal, while both VP8* and the anti-αGal antibodies stained the GGTA1-transfected cells. The VP8* and the MAb labeling did not overlap, likely due to competition for the same ligand. Nuclei were stained with DAPI. Scale bars, 20 μm.

A synthetic HBGA binding assay was then performed using the VP8* domains of both strains. The results showed strong binding of VP8* domains to the synthetic αGal HBGA (Fig. 3A). To confirm the specific binding of both P[5]-bearing strains to the αGal epitope, we examined whether the removal of αGal epitope from the synthetic Galα3Galβ4GlcNAcβ HBGA by pretreatment with α-galactosidase (GLA) was able to reduce the binding of both strains. Pretreatment with GLA reduced the binding of both VP8* domains to synthetic Galα3Galβ4GlcNAcβ HBGA (Fig. 3B).

CHO cells, which are devoid of the αGal epitope, were transfected with a sequence encoding α1,3-galactosyltransferase (GGTA1), the enzyme responsible for the synthesis of the αGal epitope (Fig. 3C). CHO cells expressing the αGal epitope allowed binding of the two P[5] VP8* protein, thus confirming that G6P[5] WC3 and reassortant G4P[5] strains recognize the αGal epitope of HBGA (Fig. 3C). However, both parent and GGTA1-transfectant CHO cells failed to allow the replication of either strains (data not shown), suggesting that expression of αGal epitope is not sufficient to establish virus replication and that other receptors and/or intracellular factors are required.

### Salivary binding assay confirmed that P[5]-bearing strains used the αGal HBGA as a ligand.

**FIG 4:**
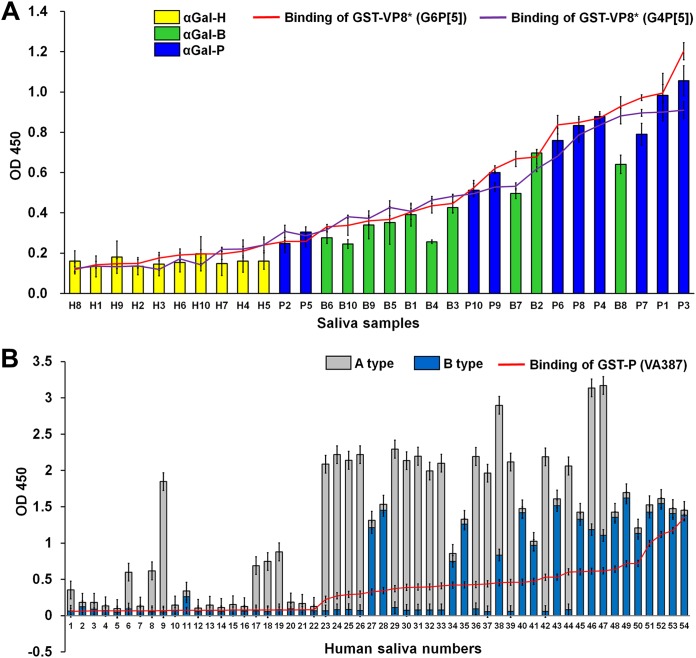
Binding of recombinant VP8* domain of bovine G6P[5] WC3 and bovine-human mono-reassortant G4P[5] strains to saliva. (A) Binding of the GST-VP8* domains was tested on a panel of human, bovine, and porcine saliva samples, using an anti-GST antibody (1:1,000 dilution), followed by the addition of an HRP-conjugated goat anti-mouse IgG antibody. The binding results to individual saliva samples were sorted by the αGal signals of individual saliva samples. A trend for correlation between the salivary αGal signals and VP8* binding levels for both P[5]-bearing strains was observed. Capital letters H, P, and B on the *x* axis identifies human (indicated by yellow bars), pig (indicated by blue bars), and cow (indicated by green bars) samples, respectively. (B) GST-P domain of norovirus strain VP387 (GII0.4) was tested as a positive control for binding to a panel of saliva samples from 54 human individuals. The A and B type signals of each individual saliva sample were sorted particularly based on the stronger intensity of A type signal, and the binding activity of the P domain to each individual saliva sample was plotted on a graph. A trend for correlation with the salivary A and B signals with the binding levels of the P domain was observed. The binding ability of recombinant GST-VP8* domains or GST-P domain was determined by the saliva-binding assay mentioned above. The binding of the saliva samples was visualized using TMB and measured at 450 nm in three independent experiments. Error bars represent means ± the SD.

**FIG 5:**
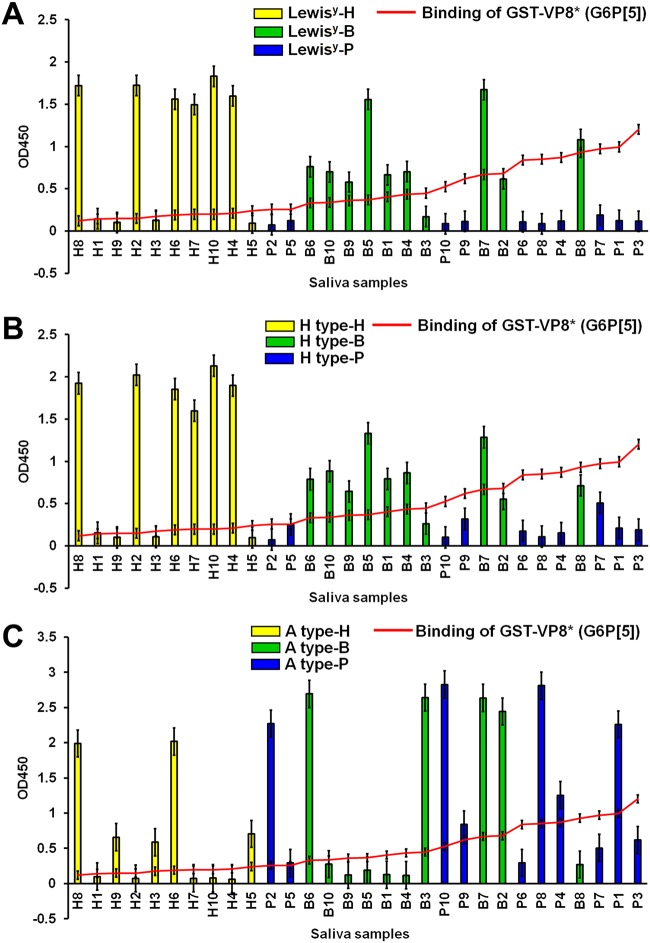
Binding activity of P[5] VP8* domains and the amount of Lewis^y^, H, and A HBGAs in saliva. Binding of the GST-VP8* domains was tested on a panel of human (capital letter H on the *x* axis, indicated by yellow bars), bovine (capital letter B on the *x* axis, indicated by green bars), and porcine (capital letter P on the *x* axis, indicated by blue bars) saliva samples, using an anti-GST antibody (1:1,000 dilution), followed by the addition of an HRP-conjugated goat anti-mouse IgG antibody. (A to C) The binding results for individual saliva samples were sorted by their Le^y^ (A), H (B), and A (C) signals. No correlation was observed between the salivary Le^y^, H, and A signals and VP8* binding levels in either P[5]-bearing strain. Binding of saliva samples was visualized using TMB, which was measured at 450 nm in three independent experiments. Error bars represent means ± the SD.

The αGal epitope is present on the cell surface and in secretions, including the saliva of pigs and cows, but it is completely absent in humans (28, 29). We determined and then sorted the αGal signals of individual saliva samples, and tested binding activity of the GST-VP8* domains on a panel of human, bovine, and porcine saliva samples arranged from low to high signals of αGal HBGA. The binding activity of VP8* domains from both strains paralleled the binding of the anti-αGal epitope in the cow and pig saliva samples; however, the binding of the VP8* domains to human saliva samples was very low (Fig. 4A). As a positive control, the P domain of the norovirus (NV) VA387 strain (GII.4) reacted strongly with human saliva samples from individuals A and B, demonstrating binding specificity (Fig. 4B). In addition, the binding activity of the VP8* domains from both strains showed no correlation with the level of Lewis^y^, H, or A HBGAs in the bovine, porcine, and human saliva samples (Fig. 5), a finding that is consistent with their lack of binding to the corresponding neoglycoconjugates. Taken together, these results clearly indicated that the VP8* domains of the G6P[5] WC3 and reassortant G4P[5] strains recognized the αGal HBGA as a ligand in cows and pigs but not in humans.

### P[5]-bearing strains were also found to use α2,6-linked SA as a receptor on permissive MA104 cells.

**FIG 6:**
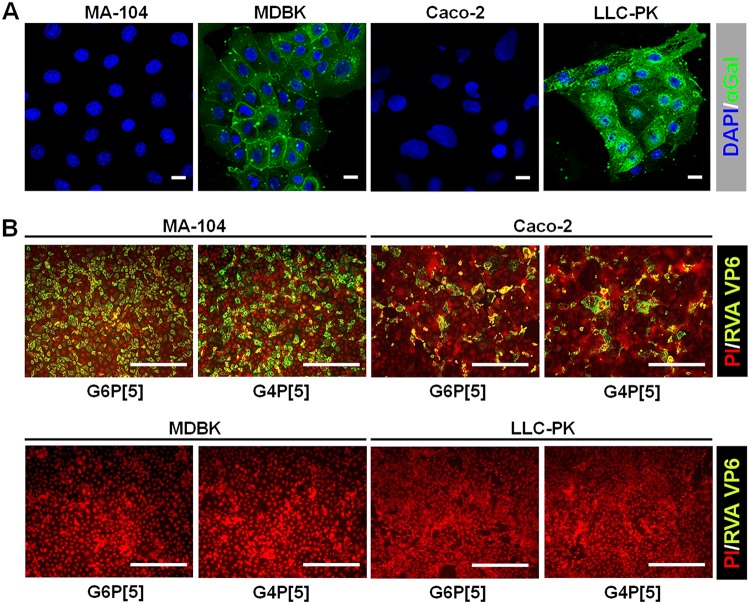
Expression of the αGal epitope and infectivity of the bovine G6P[5] WC3 and bovine-human mono-reassortant G4P[5] strains in various cell lines. (A) The expression level of the αGal epitope in Old World monkey (MA104), bovine (MDBK), human (Caco-2), and porcine (LLC-PK) cells was determined using an immunofluorescence assay with MAbs specific to the αGal epitope. Scale bars, 20 μm. (B) The infectivity of both P[5]-bearing strains (MOI, 1 FFU/cell) in the cell lines was assessed using an immunofluorescence assay with MAbs specific to the RVA VP6 protein at 15 h postinfection. All experiments were performed on three independent occasions. Scale bars, 200 μm. Panels A and B show representative images.

**FIG 7:**
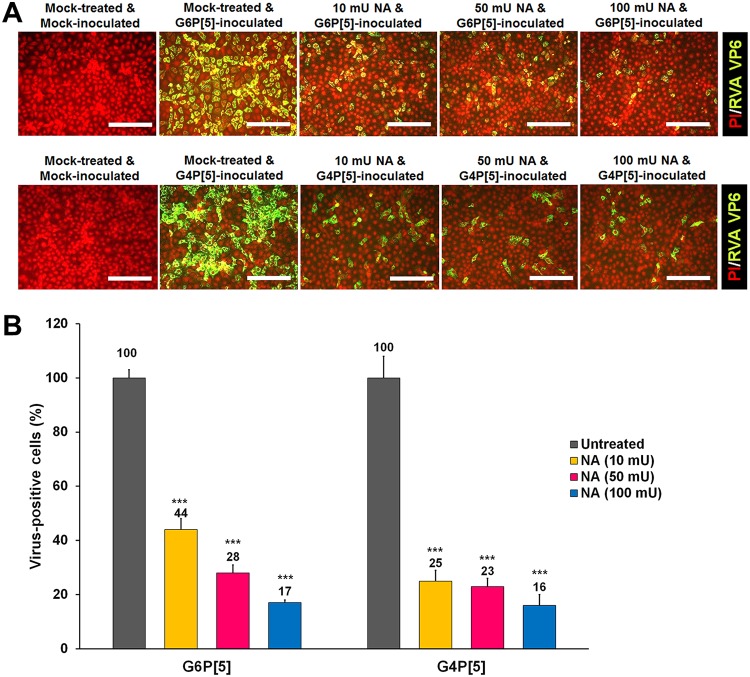
Infection of the bovine G6P[5] WC3 and bovine-human mono-reassortant G4P[5] strains requires a terminal SA. (A) The effect of pretreatment of permissive MA104 cells with neuraminidase from Vibrio cholerae on the infectivity of both P[5]-bearing RVA strains (MOI, 1 FFU/cell) was assessed using an immunofluorescence assay with MAbs specific to the RVA VP6 protein. Panel A shows one representative set of results. (B) The number of RVA antigen-positive cells following NA treatment, expressed as a percentage of mock-treated virus-infected cells, was quantified from three independent fields of view. All experiments were performed on three independent occasions. Scale bars, 200 μm. Error bars represent means ± the SD from triplicate experiments. ***, *P* < 0.001.

Although the results presented above showed that the bovine G6P[5] WC3 and mono-reassortant G4P[5] strains recognized the αGal HBGA, both strains could replicate in MA104 cells, which do not express the αGal epitope, due to the lack of an α1,3-galactosyltransferase enzyme in rhesus monkeys, the species of origin of these cells (26, 29). The αGal epitope was strongly detected on bovine (MDBK) and porcine (LLC-PK) cells, but not on Old World monkey (MA104) or human colon carcinoma-derived Caco-2 cells (Fig. 6A). Although MDBK and LLC-PK cells expressed αGal epitope, they did not allow the replication of either strain (Fig. 6B), suggesting that binding to the αGal epitope is not sufficient to make cell lines permissive to either of these strains and that other receptors and/or intracellular factors are required for the efficient entry and/or replication of these strains in these cell lines. Conversely, the replication of both strains in MA104 and Caco-2 cells (Fig. 6B) suggested that the VP8* domains of these strains may also recognize other receptors, such as internal or terminal SAs. Accordingly, we examined whether both strains were able to recognize SAs. The treatment of MA104 cells with neuraminidase (NA) from Vibrio cholerae to remove SAs decreased the infectivity of both strains in an enzyme dose-dependent manner (Fig. 7). It should be noted that even the highest NA concentration used did not fully suppress RVA infectivity (Fig. 7). Pretreating MA-104 cells with α2,3-linked SA-specific sialidase (SS) from Streptococcus pneumoniae had no significant inhibitory effects on the infectivity of either strain, regardless of the enzyme dose administered (data not shown). As expected, NA and SS reduced the infectivity of the α2,3-linked SA-dependent control virus, enterovirus 70 (EV70) J670/71 strain but did not affect the infectivity of the decay-accelerating factor (DAF)-dependent control virus, coxsackievirus B3 (CVB3) Nancy strain (data not shown). These findings strongly suggested that the bovine G6P[5] WC3 and mono-reassortant G4P[5] strains use SS-resistant and NA-sensitive SA residue(s) to infect MA104 cells.

### Preincubation with sialic acids or αGal molecule prevented infection by P[5]-bearing strains.

**FIG 8:**
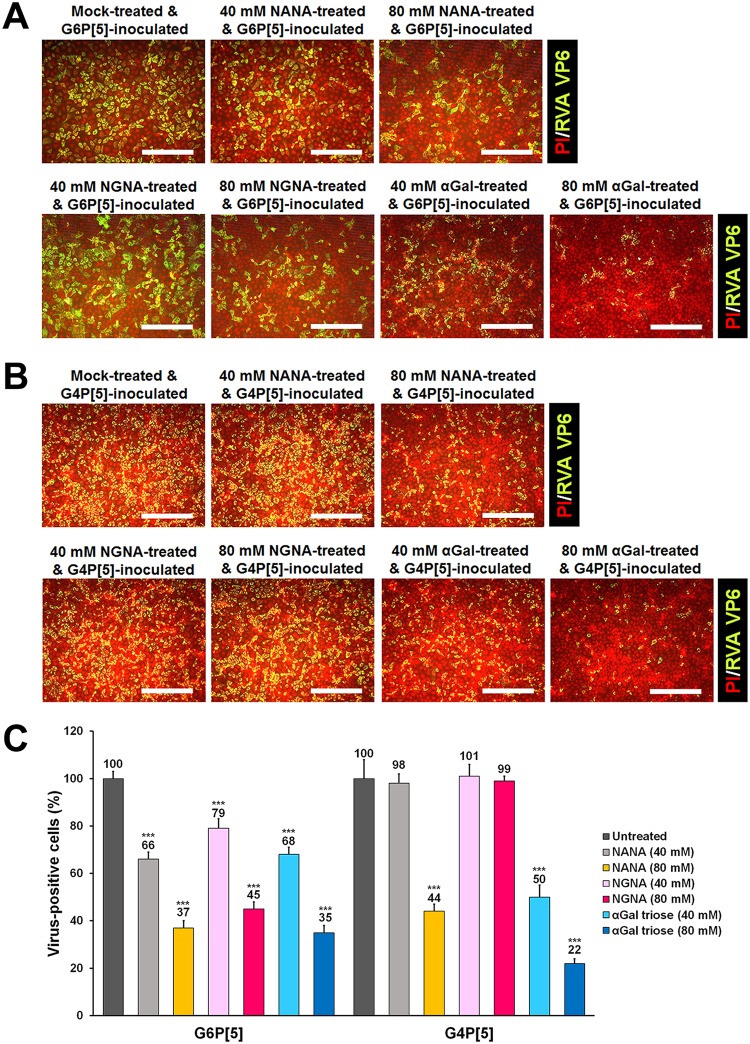
Infectivity of the bovine G6P[5] WC3 and bovine-human mono-reassortant G4P[5] strains is blocked by NANA, NGNA, or αGal. (A) The effect of preincubation of MA-104 cells with NANA, NGNA, or αGal on the infectivity of the bovine G6P[5] WC3 strain (MOI, 1 FFU/cell) was assessed using an immunofluorescence assay with MAbs specific for the RVA VP6 protein. (B) The effect of preincubation of MA-104 cells with NANA, NGNA, or αGal on the infectivity of the bovine-human mono-reassortant G4P[5] strain (MOI, 1 FFU/cell) was assessed using an immunofluorescence assay with MAbs specific for the RVA VP6 protein. (C) The number of infected MA-104 cells with both P[5]-bearing RVA strains was expressed as a percentage of the mock-treated, virus-infected control. All experiments were performed on three independent occasions. Panels A and B show representative sets of results. The scale bars in panels A and B indicate 200 μm. Error bars in panel C represent the means ± the SD from triplicate experiments. ***, *P* < 0.001.

To further determine whether SAs and αGal could act as receptors for both P[5]-bearing strains, the monosaccharides NANA and NGNA, and the αGal trisaccharide were individually premixed with bovine G6P[5] WC3 and mono-reassortant G4P[5] strains before inoculation into MA104 cells. The infectivity of both strains was blocked in a dose dependent manner when mixed with NANA (Fig. 8), while NGNA blocked the infectivity of the G6P[5] WC3 strain, but not the G4P[5] strain (Fig. 8). NANA is the predominant SA found in human and many mammalian cells, whereas NGNA is found in most nonhuman mammals (30). These results suggest that the mono-reassortant G4P[5] lost the ability to recognize the nonhuman sialic acid NGNA, indicating a possible adaptation to human glycans. Interestingly, the infection of αGal-free MA104 cells was reduced in a dose-dependent manner after preincubation of either strain with the αGal trisaccharide (Fig. 8). These findings were consistent with the use of SA as a receptor by both P[5]-bearing strains and indicated that the αGal trisaccharide interferes with a binding site required for infection, which may be the SA binding site.

### The VP8* domain of P[5]-bearing strains agglutinated rabbit erythrocytes with the αGal epitope.

To determine whether P[5]-bearing RVA strains agglutinate erythrocytes, a hemagglutination (HA) assay was performed using erythrocytes from various species (data not shown). The VP8* domains and viral particles of both bovine G6P[5] WC3 and mono-reassortant G4P[5] strains agglutinated rabbit erythrocytes but did not agglutinate erythrocytes from any other species examined (data not shown). The G11P[25] Dhaka6 strain, used as a positive control, agglutinated human blood group A erythrocytes as expected (data not shown), thus validating the HA assay. An anti-αGal antibody agglutinated rabbit erythrocytes at a high titer but did not agglutinate erythrocytes from any other species (data not shown), an observation consistent with the known high expression level of the αGal motif on rabbit erythrocytes (28). To confirm that αGal was the ligand recognized on rabbit erythrocytes, we performed the HA assay following pretreatment of rabbit erythrocytes with GLA. GLA pretreatment prevented agglutination by both strains (data not shown), indicating that both strains agglutinated rabbit erythrocytes via the αGal HBGA. Interestingly, pretreatment of rabbit erythrocytes with NA prevented their agglutination by both P[5]-bearing strains (data not shown). These results indicated that agglutination of rabbit erythrocytes by both P[5]-bearing strains involved both αGal HBGA and SA moieties.

### The P[5]-bearing strains replicated in α2,6-linked SA- and αGal-negative HIEs and in α2,6-linked SA-negative and αGal-positive BIEs.

**FIG 9:**
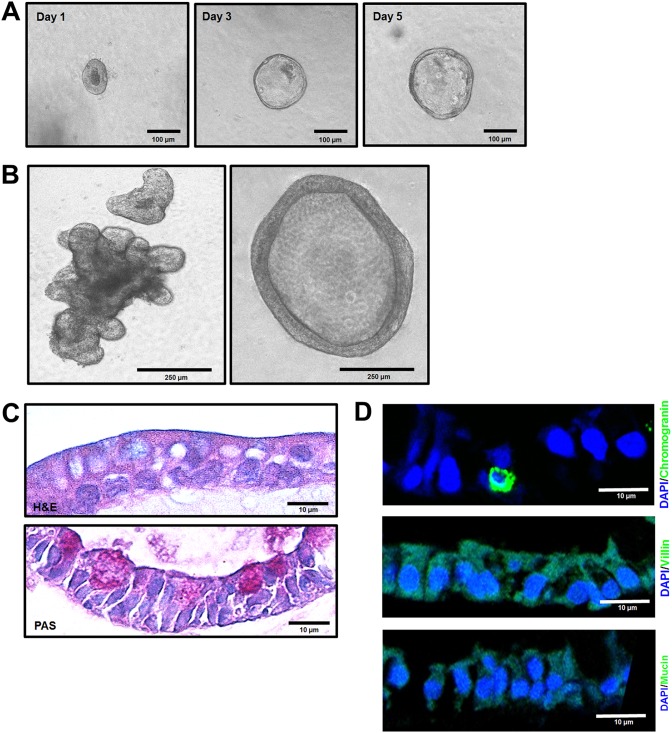
Characterization of bovine ileal enteroids. (A) Representative images of sequential bovine ileal enteroids from intestinal crypts that were grown for 1, 3, or 5 days in complete medium with growth factors. (B) After 10 days of growth in complete medium with growth factors, enteroids typically had two major morphologies: cystic (left) and multilobular (right). (C and D) Differentiated bovine ileal enteroids (3D) grown in Matrigel with a differentiation medium lacking Wnt3A, SB202190, and nicotinamide and with half the normal concentration of Noggin and R-spondin, were fixed with 4% paraformaldehyde and embedded in paraffin. (C) Thin sections were stained with H&E (upper panel), and periodic acid-Schiff (PAS, lower panel). (D) Thin sections were stained using immunohistochemistry to determine the expression levels of villin for enterocytes, chromogranin A for enteroendocrine cells, and mucin 2 for goblet cells. Nuclei were stained with DAPI.

We first characterized HIEs and bovine intestinal enteroids (BIEs). Consistent with previous reports (31, 32), when HIEs and BIEs were cultured in complete medium with growth factors, they formed multilobular or cystic structures after approximately 7 (HIEs) or 10 (BIEs) days (data not shown for human HIEs; Fig. 9A and B). Differentiated HIEs and BIEs showed scattered periodic acid-Schiff (PAS)-positive goblet cells (data not shown for human HIEs; Fig. 9C). Differentiated jejunal HIEs were positive for villin, a marker of intestinal epithelial cells, and a few lining cells were positive for chromogranin, a marker of enterochromaffin cells (data not shown). Ileal BIEs cultivated in a differentiation medium showed morphological characteristics of differentiated cells, such as enterocytes and goblet cells (Fig. 9C), and a few positive enterochromaffin cells were detected using an anti-chromogranin antibody (Fig. 9D). However, neither enterocytes nor goblet cells were identified by immunostaining for villin and mucin, respectively (Fig. 9D). In addition, antibodies specific for sucrose-isomaltase (epithelial marker) and lysozyme (Paneth cell marker) used in previous studies (31, 32) did not specifically detect their targets in HIEs or BIEs when cultivated in differentiation medium.

**FIG 10:**
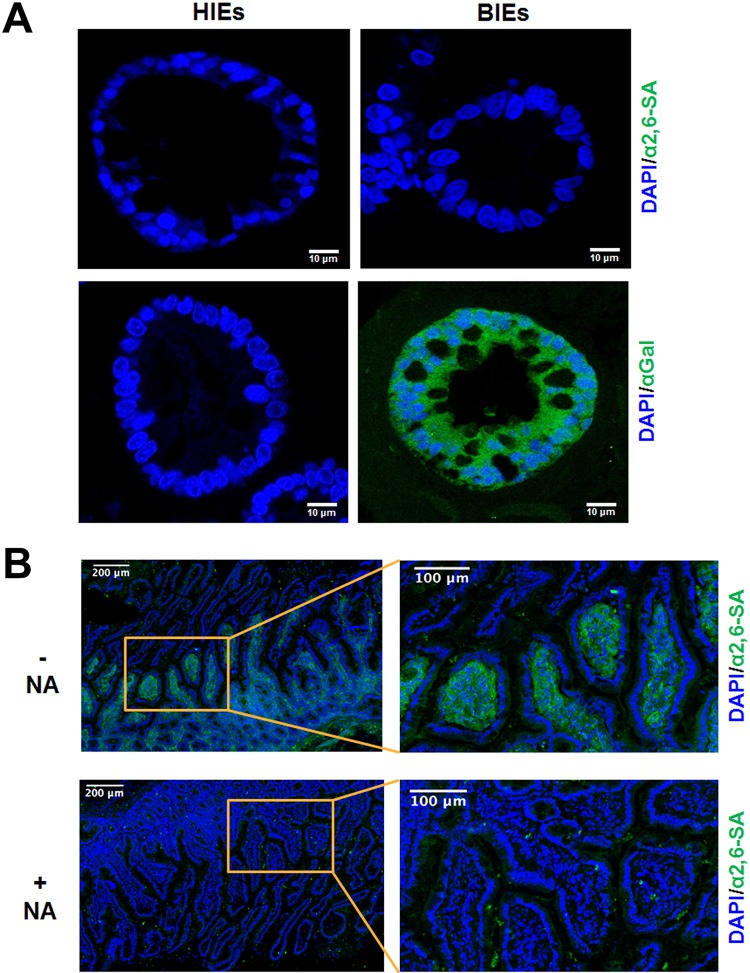
Expression of α2,6-linked SA and αGal in differentiated human and bovine jejunal enteroids and human small intestinal sections. (A) Differentiated human jejunal and bovine ileal enteroids grown in Matrigel with a differentiation medium were fixed with 4% paraformaldehyde. Thin sections of differentiated HIEs and BIEs were stained using immunohistochemistry to determine the expression levels of α2,6-linked sialic acids and the αGal epitope. Nuclei were stained with DAPI. Representative images are shown. (B) Detection of α2,6-linked SAs in the human small intestine. Frozen human duodenal tissue sections were fixed with 10% formalin and incubated with biotin-labeled SNL at 10 μg/ml in PBS, followed by incubation with DyLight 488-conjugated streptavidin (green) in the same buffer (α2,6-SA). Nuclei were stained with DAPI (blue). To determine the SA dependence of the labeling, tissue sections were pretreated with neuraminidase for 4 h (+NA). Positive control sections were preincubated with the manufacturer’s enzyme buffer only (–NA). The right panel shows higher-magnification images of the inlets from the left panel. The data are representative from two individuals.

**FIG 11:**
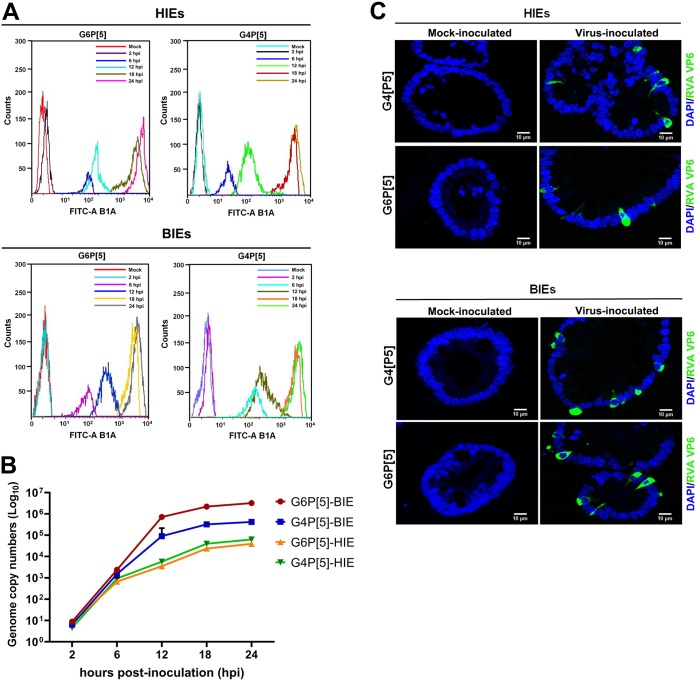
Replication of the bovine G6P[5] WC3 and bovine-human mono-reassortant G4P[5] strains in differentiated human and bovine intestinal enteroids. (A) Differentiated human jejunal and bovine ileal enteroids grown in Matrigel with a differentiation medium were infected with the bovine G6P[5] WC3 strain or the bovine and human mono-reassortant G4P[5] strain at an MOI of 10 FFU/cell for the indicated times. The infected cells were assessed using flow cytometry with an MAb against the rotavirus VP6 protein and then FITC-conjugated secondary antibodies. The data are representative from three independent experiments. (B) Differentiated HIEs and BIEs infected with the bovine G6P[5] WC3 strain or the bovine and human mono-reassortant G4P[5] strain, at an MOI of 10 FFU/cell for the indicated times, were harvested and used to determine total viral RNA by real-time RT-PCR. All experiments were performed on three independent occasions. Error bars represent means ± the SD from triplicate experiments. (C) Differentiated HIEs and BIEs grown in Matrigel with a differentiation medium were infected with the bovine G6P[5] WC3 strain or the bovine-human mono-reassortant G4P[5] strain at an MOI of 10 FFU/cell for 48 h. The cells were fixed with 4% paraformaldehyde. Thin sections were incubated with MAbs against the rotavirus VP6 protein and then FITC-conjugated secondary antibodies. Nuclei were stained with DAPI. Representative images are shown.

Differentiated HIEs were negative for both α2,6-linked SAs and αGal HBGA, whereas differentiated BIEs were negative for α2,6-linked SAs and positive for αGal HBGA (Fig. 10A). Consistent with the lack of α2,6-linked SA expression in HIEs, Sambucus nigra lectin (SNL) staining of frozen sections of human small intestine failed to detect positive epithelial cells *in situ* (Fig. 10B). Only cells from the lamina propria were specifically labeled in an NA-sensitive manner, as confirmed by the finding that NA treatment inhibited the positive staining (Fig. 10B). We then used differentiated HIEs and BIEs to examine the replication levels of both P[5] strains. Differentiated HIEs and BIEs infected with both P[5] strains showed positive detection of intracellular rotavirus antigen by flow cytometry and an increase in viral replication by qPCR (Fig. 11A and B). Nevertheless, replication in HIEs was slightly lower than that in BIEs (Fig. 11B). Scattered RVA-positive cells were observed, via confocal microscopy, in differentiated HIEs and BIEs at 18 h postinfection (Fig. 11C). These results suggested that both strains could replicate in HIEs, despite their lack of α2,6-linked SAs and αGal HBGA.

## DISCUSSION

The binding of viral particles to cell surface attachment factors and/or cellular receptor(s) regulates the initial stage of a viral infection. Thus, the different expression and distribution of attachment factors and receptor(s) plays a pivotal role in viral tissue tropism and pathogenesis (33, 34). The bovine G6P[5] RVA strains such as WC3 strain are an important animal pathogen since it has a high economic impact on the cattle industry and are a potential source of zoonotic human RVA transmission (35–38). The WC3 strain is used as the backbone of the bovine-human reassortant RotaTeq vaccine (39, 40). However, the attachment factors and/or receptors used by the VP8* domains of these strains remain unknown. Here, we demonstrated that the P[5]-bearing WC3 and bovine-human mono-reassortant strains recognized both αGal HBGA and α2,6-linked SAs as ligands.

The bovine G6P[5] WC3 strain and other bovine P[5]-bearing strains, such as UK, B-641, and 678, are reported to be NA insensitive (15). In this study, however, the infectivity of the bovine G6P[5] WC3 and G4P[5] RotaTeq vaccine strains was reduced in a dose-dependent manner by pretreating MA104 cells with NA. Moreover, identical amino acid sequences between the current and original GenBank database-deposited G6P[5] WC3 strains and between current and original GenBank database-deposited G4P[5] RotaTeq vaccine strains did not influence NA sensitivity of current viruses. In addition, one amino acid substitution at amino acid 193 (N193K) within the hemagglutination domain of VP8* domain between G6P[5] WC3 and G4P[5] RotaTeq vaccine strains (19, 26, 27) did not change the NA sensitivity of either strains, as mentioned above, indicating no influence of NA sensitivity by amino acid substitution at amino acid 193 (N193K) within the hemagglutination domain. The discrepancy between our results and previously reported data may be due to different NA treatment protocols; we pretreated MA104 cells with 10 to 100 mU/ml of NA, whereas Ciarlet et al. pretreated cells with 0.3 to 20 mU/ml of NA (15). Since the inhibitory effects of NA have also been reported in other SA-dependent viruses, such as porcine sapovirus Cowden, sapelovirus, feline calicivirus F9, and echovirus 70 J670/71 strains (41–43), we confirmed NA sensitivity using NA-sensitive EV70 and NA-independent CVB3 strains in the present study. Our data suggested that G6P[5] WC3 and G4P[5] RotaTeq vaccine strains are NA sensitive.

In addition, the infectivity of both strains was also reduced by pretreatment with the α2,6-linked SA inhibitor, SNL, but not by pretreatment with the α2,3-linked SA inhibitors SS and MAL. Taken together, these findings demonstrated that the bovine G6P[5] WC3 and human-bovine reassortant G4P[5] strains are NA sensitive and use α2,6-linked SA for the infection of MA104 cells. Since the internal SAs of cell surface carbohydrates are NA insensitive, our results cannot rule out the possibility that these strains use internal SA moieties as a ligand in a similar way to P[8] Wa, which has been shown to recognize the internal SA of ganglioside GM1 (16, 17), and Le^b^ and H type 1 HBGAs (20). Consequently, in future studies it will be necessary to fully characterize the SA-containing glycans on MA104 cells that are recognized by P[5]-bearing strains.

It has also been reported that amino acid changes in VP7 protein may influence the NA sensitivity of VP8* domain of a rotavirus strain (44). In the present study, three amino acid substitutions of VP7 proteins were detected between current G6P[5] WC3 and GenBank database-deposited original WC3 strains. However, amino acid sequences of VP7 proteins between current G4P[5] RotaTeq and GenBank database-deposited original G4P[5] strains were identical. If the three amino acid substitutions observed in the VP7 protein of current WC3 strain changed the NA sensitivity of VP8* domains from the original NA-insensitive to the current NA-sensitive G6P[5] WC strains, there should also be amino acid change(s) in the VP7 protein between current G4P[5] RotaTeq and GenBank database-deposited original G4P[5] strains because the VP8* domain of current G4P[5] RotaTeq strain was also NA sensitive like the current WC3 strain. However, no amino acid substitutions were observed between them. These suggested that VP7 proteins of G6P[5] WC3 and G4P[5] RotaTeq strains could not alter the receptor-binding specificity (NA sensitivity) of GenBank database-deposited original strains.

As expected, the preincubation of both strains with monosaccharides (NANA and/or NGNA) inhibited their infectivity in MA104 cells in a dose-dependent manner. Interestingly, the preincubation of both strains with αGal also inhibited their infectivity in αGal-free MA104 cells, suggesting that αGal binding may interfere with the α2,6-linked SA binding site. Crystallographic studies will be necessary to determine whether the α2,6-linked SA and αGal binding sites are located on the VP8* domains of both strains or whether the interference of αGal with NANA-dependent infection is indirect.

Some animal RVAs agglutinate erythrocytes using SA-containing compounds, whereas human RVAs are generally considered to be nonhemagglutinating (26). Like some caliciviruses, such as human and bovine noroviruses, rabbit hemorrhage disease virus, and Tulane virus (29, 43, 45–49), recent studies have shown that the VP8* domain of human RVAs recognizes HBGAs (19–21), inducing the agglutination of erythrocytes by binding HBGAs (21). In addition, the NA-insensitive bovine G10P[11] B223 and bovine-human reassortant G10P[11] strains recognize the type II HBGA precursor and then agglutinate type O, A, and B erythrocytes (24). P[5]-bearing strains, including bovine strains UK, B-641, WC3, and 678, are considered to be NA insensitive (15) and HA insensitive to human group O erythrocytes (26, 50). In the present study, both bovine G6P[5] WC3 and bovine-human reassortant G4P[5] strains agglutinated rabbit erythrocytes, but not the erythrocytes from any other species, which is consistent with the high expression of αGal epitope on rabbit erythrocytes (28). In addition, the HA activity of both strains on rabbit erythrocytes was inhibited by pretreatment with GLA and NA, indicating that both P[5]-bearing strains agglutinated rabbit erythrocytes by binding αGal and SA moieties (21, 23, 24).

A growing body of evidence indicates that RVAs may be transmitted between animals and humans (3, 37). However, P[5]-bearing strains do not naturally infect humans even though they are used in the RotaTeq vaccine as the bovine-human mono-reassortant P[5] strains G1P[5], G2P[5], G3P[5], and G4P[5]. The αGal antigen is present in bovine small intestine epithelial cells, while the ganglioside, GD1α, containing an α2,6-linked SA, has been detected in calf small intestine epithelial cells, suggesting that these two ligands are involved in the infection of calves which are the natural host of P[5]-bearing RVA strains (29, 51). Interestingly, bovine norovirus strains also specifically bind to αGal in the bovine small intestinal mucosa but not in human or porcine mucosa (29). This suggests the convergent adaptation of rotaviruses and noroviruses to bovine host glycans. In contrast, humans and Old World monkeys do not express αGal because they lack an α1,3-galactosyltransferase (28). Similarly, although α2,6-linked SA moieties are abundant on many cell types, where they serve as receptors for several other viruses (34, 52, 53), they have not been detected on glycolipids or N-linked glycoproteins in the gut of adult humans. They have only been found on the innermost *N*-acetylgalactosamine of O-glycans, which is unlikely to be accessible to a virus, since none have been detected when staining the human gut intestine with α2,6-linked SA-specific SNL (54–57). Therefore, the absence of both αGal and α2,6-linked SAs in the human intestine likely contributes to the lack of bovine-to-human cross-species transmission by P[5]-bearing strains.

To determine whether bovine RVAs could replicate in homologous and heterologous species, we used the novel pathophysiological model of HIEs and BIEs (31). Interestingly, we demonstrated that both the bovine G6P[5] WC3 and bovine-human mono-reassortant G4P[5] strains were able to replicate in HIEs, albeit somewhat less efficiently than in BIEs, suggesting that both strains may have some potential for zoonotic transmission. We also demonstrated that the differentiated HIEs, which allowed the replication of both P[5]-bearing strains, did not express α2,6-linked terminal SAs or αGal. It therefore appears that these ligands are dispensable for infection of human cells by the bovine G6P[5] WC3 and bovine-human mono-reassortant G4P[5] strains *in vitro*. This is similar to the situation encountered with the human P[8] strains, which can infect cells depleted of fucosylated HBGA ligands *in vitro* (21). The importance of the HBGA ligands of P[8]-bearing strains *in vivo* is, nonetheless, well documented since so-called nonsecretor children, who present inactivating mutations in the *FUT2* gene, seldom develop symptomatic gastroenteritis caused by P[8]-bearing strains and seroconvert less efficiently than secretor children after vaccination (18, 19, 58–60). Likewise, the lack of αGal and α2,6-linked SA on human epithelial cells could limit the extent of infection caused by the P[5]-bearing strains in the RotaTeq vaccine.

The infection of HIEs by P[5]-bearing strains suggested that additional or alternative receptors and mechanisms of entry can be used for infection *in vitro*. Earlier studies have shown that the RRV mutant strain, nar3, binds directly to the cell through the VP5* domain of VP4 protein (61). Likewise, integrins, hsp70, and tight-junction proteins have been suggested as alternative RVA receptors (5). Such attachment factors and/or receptors may be used by the P[5]-bearing strains to initiate the infection of human cells. Therefore, whether the VP5* domain of P[5] VP4 protein directly binds to α2,6-linked SA- and αGal-free HIEs and human intestinal epithelial cells without prior attachment of VP8* domain should be addressed in future studies. This could be demonstrated by using several sophisticated tools, including recently developed reverse genetics to incapacitate individual genes or to introduce particular amino acid substitutions (62–64).

In conclusion, we demonstrated that the bovine G6P[5] WC3 and human-bovine mono-reassortant G4P[5] strains both use α2,6-linked SAs and αGal as ligands. This likely explains the absence of natural human infections by P[5]-bearing strains, since neither ligand is present in the epithelium of the human small intestine. However, both strains could replicate in HIEs lacking α2,6-linked SAs and αGal, suggesting that P[5]-bearing strains can initiate, albeit less efficiently, the infection of human cells through alternative ligands or receptors and thus may have some potential for zoonotic transmission.

## MATERIALS AND METHODS

### Cells and viruses.

MA104 (African green monkey kidney) cells obtained from the American Type Culture Collection (ATCC) were cultured in alpha minimum essential medium supplemented with 5% fetal bovine serum (FBS), 100 U/ml penicillin, and 100 μg/ml streptomycin. Porcine kidney LLC-PK, human colorectal adenocarcinoma Caco-2, Madin-Darby bovine kidney (MDBK), human cervical cancer HeLa cells, and human lung fibroblast WI-38 cells were all purchased from the ATCC and were cultured in Eagle medium (EMEM) supplemented with 10% FBS, 100 U/ml penicillin, and 100 μg/ml streptomycin. Human embryonic kidney 293T cells stably expressing R-spondin 1 (Trevigen, Gaithersburg, MD) or Noggin (kindly provided by G. R. van den Brink, Hubrecht Institute, The Netherlands) were cultured in flasks with Dulbecco modified Eagle medium (DMEM; without arginine or lysine) supplemented with 10% FBS, GlutaMAX (1×), HEPES (1×), penicillin (100 U/ml), and streptomycin as described previously (31, 32). The bovine RVA G6P[5] WC3 strain was kindly provided by J. Matthijnssens (University of Leuven, Leuven, Belgium). The bovine-human mono-reassortant G4P[5] strain, a component of the RotaTeq pentavalent RVA vaccine, was a generous gift from S. J. Lee (SK Chemicals, Seongnam-si, Republic of Korea). The other RVA strains, including bovine G6P[1] NCDV, canine G3P[3] CU-1, enterovirus 70 (EV70) J670/71, and coxsackievirus B3 (CVB3) Nancy, were all purchased from the ATCC. Before inoculation of cells, the RVA strains were preincubated with 10 μg/ml of trypsin for 1 h, and then the RVA strains, EV70, and CVB3 were propagated in MA104, Caco-2, and WI38 cells, respectively (41, 65, 66). Viral titers were assessed using a cell culture immunofluorescence assay with monoclonal antibodies (MAbs) specific for each virus, as described below, and expressed as fluorescence focus units (FFU)/ml.

### Sequencing, expression, and purification of the GST-VP8* domain and GST-P particle and sequencing of VP7 genes.

The full-length sequences of VP8* corresponding to amino acids 1 to 231 of bovine G6P[5] WC3 and G6P[1] NCDV, canine G3P[3] CU-1, and bovine-human mono-reassortant G4P[5] RVA strains were amplified from infected cell cultures using an reverse transcription-PCR (RT-PCR) assay with specific primers (Table 1), as described previously (20). The cDNA encoding the full-length VP8* sequences of G11P[25] Dhaka6 (GenBank accession no. GU199520) and the P domain of human norovirus genogroup II genotype 4 strain VA387 (GenBank accession no. AAK84679) were synthesized (Bioneer, Daejeon, South Korea) (19, 67). Each amplicon, as well as the cDNA encoding the Dhaka6 VP8* domain or the VA387 P domain, was cloned into a pGEX4T-1 expression vector with an N-terminal glutathione *S*-transferase (GST) tag (GE Healthcare Life Sciences, Pittsburgh, PA). The GST-VP8* proteins were expressed and purified as described previously (20).

**TABLE 1:** Primers used for amplification of the RVA VP8*, VP7, and NV P domain coding regions

Strain	Sequence (5′–3′)^*a*^	Construct generated	Restriction enzyme site	Sense
G6P[1] NCDV	GTGGATCCATGGCTTCACTCATTTATAGAC	VP8* full-length	BamHI	Positive
	GCCTCGAGTCATCTCGTATTTTGTATTGGTGG		XhoI	Negative
G6P[5] WC3	GTGGATCCATGGCTTCGCTCATATACAG	VP8* full-length	BamHI	Positive
	GCCTCGAGTCATCTGGTGTTTTGGATTG		XhoI	Negative
	GGCTTTAAAAGAGAGAATTTCCGT	VP7 full-length		Positive
	GGTCACATCATACAATTCTAATCT			Negative
G4P[5] RotaTeq	GTGGATCCATGGCTTCGCTCATATACAG	VP8* full-length	BamHI	Positive
	GCCTCGAGTCATCTGGTGTTTTGGATTG		XhoI	Negative
	GGCTTTAAAAGAGAGAATTTCCGT	VP7 full-length		Positive
	GGTCACATCAAACAGTTCTATTTT			Negative
G3P[3] CU-1	GTGGATCCATGGCTTCGCTCATTTATAGAC	VP8* full-length	BamHI	Positive
	GCCTCGAGTCATCTTGTATTTTGAATCGGTGG		XhoI	Negative
G11P[25] Dahaka	GTGGATCCATGGCTTCGTTAATTTACAGAC	VP8* full-length	BamHI	Positive
	GCCTCGAGTCAACGTGTATTCTGAATTGGTGG		XhoI	Negative
VA387 (GII.4)	GCACGGATCCTCAAGAACTAAACCATTCACC	P-CDCRGDCFC	BamHI	Positive
	GCATGCGGCCGCTTAGCAAAAGCAATCGCCACGGCAATC GCA TAATGCACGTCTGCGCCCCGC		NotI	Negative

a Underlining indicates the restriction enzyme site described in column 4.

The full-length open reading frame sequences of VP7 gene corresponding to amino acids 1 to 326 of the bovine G6P[5] WC3 and bovine-human mono-reassortant G4P[5] RVA strains were amplified from infected cell cultures using an RT-PCR assay with specific primers (Table 1), as described previously (68). Each purified VP7 amplicon by a QIAEX II gel extraction kit (Qiagen, Valencia, CA) was ligated into the pGEM-T Easy vector systems (Promega, Madison, WI) and subcloned into home-made DH5α competent cells, as described previously (68).

To determine whether the VP8* domain and VP7 genes of the G6P[5] WC3 and G4P[5] RotaTeq vaccine strains used in this study have amino acid mutations compared with their GenBank database-deposited original strains, the DNA of plasmids encoding the VP8* domains and VP7 genes was sequenced using an ABI 3700 (Applied Biosystems, Foster City, CA). Sequence comparisons of VP8* and VP7 genes were done using the EMBL-EBI sequence analysis tools.

### Sequences and phylogenetic analyses of the full-length amino acid sequences of VP8* domains and VP7 protein.

The full-length amino acid sequences of VP8* and VP7 of the bovine G6P[5] WC3 and human-bovine mono-reassortant G4P[5] strains used in this study were compared with those of GenBank database-deposited original bovine G6P[5] WC3 _(GenBank accession no. AY050271 for VP8* and GenBank accession no. AY050272.1 for VP7) and mono-reassortant G4P[5] RotaTeq vaccine (GenBank accession no. GU565090 for VP8* and GenBank accession no. GU565090.1 for VP7) strains, using the DNA Basic module (DNAsis MAX, Alameda, CA) according to the manufacturer’s instructions. The functional moieties of each region, including the hemagglutination (HA) domain, known to bind cell surface sialic acids (SAs), and HBGA binding sites were adapted from other reports (19, 26). Phylogenetic analysis of the full-length VP8* amino acid sequence was conducted with strains representative of the five P genogroups (P[1] to P[5]) using MEGA (v4.1), and a dendrogram was constructed using the neighbor-joining method as described previously (21, 69).

### Reagents and antibodies.

*N*-Acetylneuraminic acid (NANA; Sigma-Aldrich, St. Louis, MO), *N*-glycolylneuraminic acid (NGNA; Sigma-Aldrich), free αGal trisaccharide (carbohydrate synthesis), MAL (Sigma-Aldrich), SNL (Sigma-Aldrich), biotin-labeled SNL (Vector Laboratories, Burlingame, CA), NA from Vibrio cholerae (Sigma-Aldrich), or Clostridium perfringens (New England Biolabs, Ipswich, MA), SS from Streptococcus pneumoniae (Prozyme, Hayward, CA), and GLA from green coffee beans (Sigma-Aldrich) were dissolved in phosphate-buffered saline (PBS; pH 7.2). Alexa Fluor 594 (AF594) succinimidyl ester, purchased from Molecular Probes (Eugene, OR), was dissolved in dimethyl sulfoxide (DMSO). G418 was purchased from Gibco (Paisley, UK). For the maintenance and/or differentiation of HIEs and BIEs, the following reagents were purchased: GlutaMAX-1, N2 supplement, B27 supplement, mouse recombinant epithelial growth factor, HEPES, and advanced EMEM/F12 (all from Invitrogen, Carlsbad, CA), phenol-free Matrigel (Corning, NY); *N*-acetylcysteine, SB202190, nicotinamide, and [Leu15]-Gastrin I (all from Sigma-Aldrich); A-83-01 (Tocris, Bristol, UK); WNT3a (ATCC); R-spondin (Trevigen, Gaithersburg, MD); Y-27632 (APExBIO, Houston, TX); and primocin (InvivoGen, San Diego, CA).

Biotin-conjugated oligosaccharide-polyacrylamides (PAAs)—including Lewis antigens (Le^a^, Le^b^, Le^x^, and Le^y^); type H, type A, and type B trisaccharides; αGal trisaccharides; and sLe^a^ and sLe^x^ tetrasaccharides—were purchased from GlycoTech, Gaithersburg, MD) (Table 2). The following antibodies were used in this study: anti-RVA VP6 capsid MAb (Median Diagnostics, Gangwon, South Korea), anti-EV70 capsid MAb (GeneTex, Irvine, CA), anti-CVB3 capsid MAb (Millipore, Burlington, MA), anti-αGal MAb (Enzo Life Science, Farmingdale, NY), biotinylated anti-SNL MAb (Vector Laboratories), MAbs against each HBGA molecule (Le^a^, Le^b^, Le^x^, Le^y^, H-1, H-2, A, and B; BioLegend, San Diego, CA), villin (Avivasysbio, San Diego, CA), chromogranin A and lysozyme (Novus Biologicals, Littleton, CO), sucrose-isomaltase (Santa Cruz, Dallas, TX), and mucin 2 (Abcam, Cambridge, MA). The fluorescein isothiocyanate (FITC)-conjugated donkey anti-rabbit IgG polyclonal antibody, FITC-conjugated goat anti-mouse IgG polyclonal antibody, horseradish peroxidase (HRP)-conjugated streptavidin, and HRP-conjugated goat anti-rabbit IgG and anti-mouse IgG were obtained from Jackson Immuno Research Lab (West Grove). The Alexa Fluor 488-conjugated goat anti-rabbit IgG polyclonal antibody was purchased from Life Technologies (Eugene, OR). Streptavidin-phycoerythrin (PE) and streptavidin-DyLight 488 conjugates were purchased from Vector Laboratories (Burlingame, CA). SlowFade Gold antifade reagent with DAPI (4′,6′-diamidino-2-phenylindole) was obtained from Invitrogen.

**TABLE 2:** Panel of synthetic histo-blood group antigens used in this study

Histo-blood group antigen	Oligosaccharide structure and type of synthetic spacer
Le^a^	Galβ1-3(Fucα1-4)GlcNAc-PAA-biotin
Le^b^	Fucα1-2Galβ1-3(Fucα1-4)GlcNAc-PAA-biotin
Le^x^	Galβ1-4(Fucα1-3)GlcNAc-PAA-biotin
Le^y^	Fucα1-2Galβ1-4(Fucα1-3)GlcNAc-PAA-biotin
Blood type H	Fucα1-2Galβ-PAA-biotin
Le^d^ (H type 1)	Fucα1-2Galβ1-3GlcNAc-PAA-biotin
H (type 2)	Fucα1-2Galβ1-4GlcNAc-PAA-biotin
Blood type A	GalNAcα1-3Galβ-PAA-biotin
Blood type B	Galα1-3Galβ-PAA-biotin
Blood type A (tri)	GalNAcα1-3(Fucα1-2)Galβ-PAA-biotin
Blood type B (tri)	Galα1-3(Fucα1-2)Galβ-PAA-biotin
Sialyl Le^a^	Neu5Acα2-3Galβ1-3(Fucα1-4)GlcNAc-PAA-biotin
Sialyl Le^x^	Neu5Acα2-3Galβ1-4(Fucα1-3)GlcNAc-PAA-biotin
αgal (tri)	Galα1-3Galβ1-4GlcNAcβ-PAA-biotin

### Synthetic HBGA binding assay.

The synthetic oligosaccharide-based HBGA binding assay was carried out as described previously (20, 42, 70). Briefly, 96-well microtiter plates were coated with recombinant GST-VP8* and GST-P domain proteins at 10 μg/ml and incubated at 4°C overnight. After blocking with PBS containing 5% bovine serum albumin (PBS-BSA) at 37°C for 1 h, biotinylated PAA-conjugated oligosaccharides (5 μg/ml) was added, and plates were incubated at 4°C overnight. Bound oligosaccharides were detected using HRP-conjugated streptavidin. The plates were washed five times with PBS (150 μl) containing 0.1% Tween 20 (PBS-Tween 20). The signal intensity was visualized using 3,3′,5,5′-tetramethylbenzidine (TMB; Komabiotech, Seoul, Korea), and the optical density (OD) at 450 nm was read using a microplate reader (Thermo Fisher Scientific) according to the manufacturers’ instructions.

### Saliva binding assay.

Saliva samples from 54 human individuals, 10 cows, and 10 pigs were selected from the archives of the Saliva Registry of the Laboratory of Veterinary Pathology (College of Veterinary Medicine, Chonnam National University). Before the saliva binding assay was performed, the amount of each HBGA was determined as described previously (72). Briefly, boiled saliva samples were diluted in PBS (1:20 or 1:1,000 dilution) and then coated onto microtiter immunoplates (Thermo Fisher Scientific) at 4°C overnight. After blocking with PBS-BSA at 37°C for 1 h, MAbs specific to H1, H2, Le^a^, Le^b^, Le^x^, Le^y^, type A, and type B (1:200 to 1:300 dilution) and αGal (1:5 dilution) HBGAs were added. After incubation for 1 h at 37°C, HRP-conjugated goat anti-mouse anti-IgG or IgM was added. After each step, the plates were washed five times with PBS. The color reaction was performed as described above.

A saliva binding assay was used to detect the binding of recombinant GST-VP8* and GST-P domain proteins by modifying a previously described method (72, 73). Briefly, boiled saliva samples were diluted at a ratio of 1:20 and then coated onto 96-well microtiter plates, followed by incubation at 4°C overnight. After blocking with PBS-BSA at 37°C for 1 h, recombinant GST-VP8* and GST-P domain proteins (10 μg/ml) were added, followed by incubation for a further 1 h at 37°C. Bound target proteins were detected using an anti-GST antibody (1:1,000 dilution), followed by the addition of HRP-conjugated goat anti-mouse IgG antibody. The plates were washed five times with PBS-Tween 20. The signal intensity was visualized using TMB kit (Komabiotech), and the OD at 450 nm was read using a microplate reader (Thermo Fisher Scientific) according to the manufacturers’ instructions.

To determine the target αGal epitope for the VP8* domains of both bovine G6P[5] WC3 and human-bovine mono-reassortant G4P[5] strains, αGal epitopes were removed from the bovine, human, and porcine saliva samples, and then the binding specificity of the VP8* domain was analyzed as described previously (24, 29, 70). Briefly, boiled saliva samples were diluted at a ratio of 1:20 and then coated onto 96-well microtiter plates and incubated at 4°C overnight. The plates were washed three times with PBS-Tween 20 and then incubated with 100 μl of α-galactosidase solution (4 mU/ml) for 48 h at 37°C. After blocking with PBS-BSA at 37°C for 1 h, the saliva binding assay was performed as described above. The signal intensities were visualized using a TMB kit, as described above.

### Treatment of cells with chemicals and enzymes.

MA104 cells were pretreated with various concentrations of NA or SS (10, 50, or 100 mU) for 1 h at 37°C. The lectins MAL and SNL were used at 100 μg/ml for 1 h at 4°C. NANA, NGNA, and the αGal trisaccharide (Galα3Galβ4GlcNAcβ) were preincubated with viruses (40 or 80 mM) for 1 h at 37°C. After pretreatment, the cells were washed three times with PBS. The infection assay was carried out for each virus as described below. Mock and control treatments were performed at the same time.

### VP8* protein binding assay using CHO cells transfected with GGTA1.

CHO cells were transfected with or without the PCR3.1 eukaryotic expression vector encoding the complete rat *Ggta1* gene coding sequence as described previously (28, 29). Briefly, CHO cells maintained in DMEM/F-12 supplemented with 10% FBS, 2 mM l-glutamine, free nucleotides (10 μg/ml), 100 U/ml penicillin, and 100 μg/ml streptomycin were transfected with rat *Ggta1* using Lipofectamine (Invitrogen) according to the manufacturer’s instructions. Stable transfectants were obtained by selection with 0.5 mg/ml G418. Cells were then cultured in the presence of 0.1 mg/ml G418, passaged at confluence after dispersal with 0.025% trypsin in 0.02% EDTA (which was incubated for 10 min), and routinely checked for mycoplasma contamination using Hoechst 33258 (Sigma-Aldrich) labeling.

Before binding assay, GST-VP8* domain was labeled with AF594 as described previously with slight modification (70). Briefly, each VP8* domain (10 mg at 1 mg/ml^−1^) in 0.1 M sodium bicarbonate buffer (pH 8.3) was labeled with a 0.1-fold molar concentration of AF594 succinimidyl ester (1 mg at 1 mg ml^−1^ in DMSO). Each mixture was thoroughly vortexed for 30 s and incubated for 1 h at room temperature with continuous stirring. Labeled proteins were purified as described above (20). The concentrations of the purified AF594-labeled VP8* domains were determined using a BCA protein assay kit (Pierce, IL) according to the manufacturer’s instructions.

To determine the binding of each VP8* domain of G6P[5] WC and G4P[5] RotaTeq strains, the immunofluorescence assay was performed as described previously with slight modification (70, 71). Briefly, the confluent mock-transfected parent or *GGTA1*-transfected CHO cells grown on eight-chamber slides were coincubated with the FITC-conjugated anti-αGal MAb (1:100 dilution) and the AF594-labeled VP8* proteins of either the bovine G6P[5] WC3 or human-bovine mono-reassortant G4P[5] strain at 10 μg/ml at room temperature for 30 min, washed with PBS containing 0.1% newborn calf serum (PBS-NCS), and then fixed with 4% paraformaldehyde in PBS for 1 h. The cells were then permeabilized by the addition of 0.2% Triton X-100 and washed with PBS-NCS. After washing, the cells were strained for the nuclei and analyzed using an immunofluorescence assay as described below.

### Infectivity assay.

An infectivity assay of the various cell lines was carried out according to methods described previously (41, 71, 74) with slight modifications. Briefly, confluent monolayers of cells in 8-well chamber slides were pretreated with various inhibitors or enzymes as described above. Mock or treated cells were infected with EV70, CVB3, or trypsin-pretreated RVAs at a multiplicity of infection (MOI) of 1 FFU/cell, followed by incubation at 37°C for 1 h. The cells were washed three times with PBS, and a maintenance medium was added. Cells were incubated for 15 h (RVAs), 10 h (EV70 J670/71 strain), or 5 h (CVB3 Nancy strain) at 37°C prior to fixing with 80% cold acetone in PBS (pH 7.4) or 4% paraformaldehyde. Cells fixed with paraformaldehyde for detection of the RVA VP6 protein were permeabilized by the addition of 0.2% Triton X-100 in PBS. They were then analyzed using an immunofluorescence assay as described below.

### Hemagglutination assay.

An HA assay was performed using erythrocytes from animals and humans according to methods described previously (24, 41) with slight modifications. Briefly, blood from rabbits, cows, pigs, chickens, and mice was obtained from the College of Veterinary Medicine, Chonnam National University, while adult human blood samples corresponding to the blood types O, A, and B were donated by volunteers (human ABO at Chonnam National University Hospital. Each blood sample was centrifuged for 10 min at 500 × *g*, and 0.5% suspensions of each erythrocyte sample were prepared in PBS (pH 7.2) without Ca^2+^ (Lonza; pH 7.4). Bovine G6P[5] WC3, bovine-human mono-reassortant G4P[5], GST-VP8* of RV strains, and GST alone were serially diluted using PBS in V-shape 96-well microtiter plates (Greiner Bio-One) and mixed with an equal volume of each erythrocyte suspension. The suspensions were then incubated at 4°C overnight, and the HA titer was recorded as the highest dilution of sample that resulted in complete HA. The GST-VP8* of human RVA strain G11P[25], known to hemagglutinate type A erythrocytes, was included as a positive control. The assay was also performed with GST alone to rule out false positivity due to the GST tag.

### Hemagglutination inhibition assay.

To determine the effect of treating erythrocytes with NA on HA, rabbit erythrocytes were treated with Vibrio cholerae NA or with GLA according to methods described previously (24) with slight modifications. Briefly, 0.5% rabbit erythrocytes in PBS (pH 6.0 or pH 7.4) were treated with NA (0 or 25 mU) for 2 h at 37°C or GLA (50 mU) for 6 h at 37°C. After incubation, the cells were washed, and the HA assay was carried out as described above.

### Human jejunal and bovine ileal enteroid cultures.

Human intestinal enteroids from a jejunal biopsy sample were kindly provided by M. Estes, (Department of Molecular Virology and Microbiology, Baylor College of Medicine, Houston, TX) and grown as multilobular, three-dimensional (3D) cultures in Matrigel and maintained as described previously (31). Bovine ileal enteroids from ileal specimens were obtained from colostrum-deprived neonatal calves. The BIEs prepared from the tissue samples were grown as multilobular, 3D cultures in Matrigel and maintained as described previously (31). To characterize differentiated BIEs, HIEs, and RVA infections, the HIEs and BIEs were grown in 48-well microtiter plates using differentiation medium without Wnt3A, SB202190, and nicotinamide, as well as half the concentration of Noggin and R-spondin, as described previously (31, 32).

To ensure the same viral titer was inoculated into the differentiated HIEs and BIEs, the number of differentiated cells in the HIE and BIE samples was calculated as described previously (31). To dissociate the HIEs and BIEs into a single-cell suspension, Accutase cell dissociation solution (BD Biosciences) was transferred into each well of a 48-well plate and incubated for 30 min at 37°C; then, the number of cells was counted using a hemocytometer. The MOI of the HIE and BIE samples was calculated as the viral input amount/total number of cells (31). The total number of cells within a Matrigel plug of HIEs or BIEs ranged between 100,000 and 150,000 or between 200,000 and 250,000, respectively.

### Characterization of HIEs and BIEs.

To characterize whether HIEs and BIEs were differentiated, hematoxylin and eosin (H&E) staining, PAS straining, and immunohistochemistry (IHC) were performed as described previously (31, 32). Briefly, HIEs and BIEs grown as multilobular, 3D cultures in Matrigel were fixed with 4% paraformaldehyde for 30 min at 4°C, transferred into 2% agarose gel for sectioning, manually processed, and embedded in paraffin. Serial 3-μm sections were used for H&E straining, PAS staining, and IHC.

To characterize the differentiated enteroid cells, deparaffinized and rehydrated serial 3-μm sections were washed with PBS, and then heat-induced antigen retrieval was performed using 10 mM citrate buffer (pH 6.0). Antibodies against sucrose-isomaltase (1:100 dilution), villin (1:100 dilution) for differentiated enterocytes, chromogranin (1:100 dilution) for enteroendocrine cells, mucin 2 (1:500 dilution) for goblet cells, and lysozyme (1:100 dilution) for Paneth cells were added (31, 32). To determine the expression levels of α2,6-linked SAs and αGal in the differentiated HIEs and BIEs, serial 3-μm sections of paraffin embedded HIEs and BIEs were incubated at 4°C overnight with MAbs against α2,6-linked SAs (1:100 dilution) and αGal (1:10 dilution). After two washes with PBS, the cells were incubated with FITC-conjugated secondary antibodies for 1 h at room temperature. To stain the nuclei, chambers were mounted with SlowFade Gold antifade reagent containing 1× DAPI solution (Invitrogen), and cells were examined using confocal microscopy.

### Comparison of viral infectivity in HIEs and BIEs.

The viral infectivity of both bovine G6P[5] WC3 and bovine-human mono-reassortant G4P[5] strains in HIEs and BIEs was determined as described previously (31, 32). Briefly, HIEs and BIEs differentiated for 3 to 4 days were harvested by washing with cold CMGF(–), and then equal amounts were transferred into 5-ml round-bottom polystyrene tubes (Falcon, Corning, NY). To enhance the infectivity of RV strains, 10 μg/ml of trypsin was added, and samples were incubated for 30 min at 37°C. MA104 cell lysates were also pretreated with trypsin, serving as mock-treated controls. Both HIEs and BIEs were vigorously pipetted 10 to 20 times with a P200 pipette to disperse them for optimal viral exposure. The HIEs and BIEs were inoculated at an MOI of 1 FFU/ml, and viral adsorption proceeded for 2 h in a complete medium containing 0.2 mg/ml pancreatin (Sigma-Aldrich) without growth factors. The viral inoculum was removed by centrifugation, and then the enteroids were washed, suspended in differentiation medium, and incubated for various periods of time. HIEs and BIEs were fixed and sectioned as described above in order to examine the infected cells using an immunofluorescence assay. Alternatively, HIEs and BIEs were frozen and thawed three times to determine viral genome copy numbers using real-time RT-PCR, or were dispersed into a single-cell suspension as described above, to characterize the infected cells using flow cytometry.

### Immunofluorescence assay.

An immunofluorescence assay was performed as previously reported (31, 41, 71), with slight modifications. Briefly, acetone- or paraformaldehyde-fixed cells grown on 8-well chamber slides were washed with PBS. Anti-RV VP6 protein (1:100 dilution), anti-EV70 capsid (1:10 dilution), anti-CVB3 (1:500 dilution), anti-α2,6-SA (1:100 dilution), and anti-αGal (1:5 dilution) MAbs were added. Chamber slides were incubated at 4°C overnight or at 37°C for 1 h; the cells were then washed three times with PBS-NCS, and FITC- or Alexa 594-conjugated secondary antibodies (1:100 dilution) were added. To stain the nuclei, chambers were mounted with SlowFade Gold antifade reagent containing 1× DAPI solution (Invitrogen) or stained with propidium iodide; the cells were then examined using a fluorescent standard or confocal microscopy.

### Flow cytometry analysis.

To characterize RVA-infected HIEs or BIEs, flow cytometry analysis was performed as described previously (31), with slight modifications. Briefly, single-cell suspensions were prepared as described above, fixed with 4% paraformaldehyde, permeabilized with Triton X-100, and incubated for 30 min at 4°C with anti-RV VP6 protein MAb (1:5,000 dilution). Cells were washed with PBS containing 1% FBS and then incubated for 30 min at 4°C with Alexa Fluor 488-conjugated donkey anti-mouse antibody (Invitrogen; 1:2,000 dilution). After further washing with PBS containing 1% FBS, flow cytometry acquisition was performed using a MACSQuant Analyzer 10 (MACS Miltenyi Biotec, North Rhine-Westphalia, Germany), and data were analyzed using FlowLogic software (Inivia Technologies, Victoria, Australia). The single-cell population was gated by doublet discrimination, and 10,000 events were evaluated.

### Real-time RT-PCR.

To quantify the genome copy number of RVAs, real-time RT-PCR was carried out as described previously (42, 74), with some minor modifications. Briefly, total RNA was extracted from the lysates of HIEs and BIEs as described above, using the QIAmp Viral RNA minikit (Qiagen) according to the manufacturer’s instructions. The viral genome copy number was determined using one-step SYBR green real-time RT-PCR with primer pairs specific for the RVA VP6 gene as described previously (42, 74). The total volume of each reaction mixture was 20 μl, containing 4 μl of RNA template (1 μg), 10 μl of SensiFast SYBR Lo-ROX One-Step mixture (Bioline, Quantace, London, UK), 0.8 μl each of forward and reverse primers (10 μM), 0.2 μl of reverse transcriptase (Bioline), 0.4 μl of RiboSafe RNase inhibitor (Bioline), and 3.8 μl of RNase-free water. Real-time RT-PCR was performed using a rotor-gene real-time amplification system (Corbett Research, Mortlake, Australia) under the following conditions: reverse transcription at 50°C for 30 min, activation of the hot-start DNA polymerase at 95°C for 10 min, followed by 40 three-step cycles of 95°C for 15 s, 50°C for 30 s, and 72°C for 20 s. Viral RNA was quantified using the standard curve of serial 10-fold dilutions of the cRNA generated by reverse transcription of the *in vitro*-transcribed control RNA (RVA VP6 gene). The threshold was automatically defined in the initial exponential phase to reflect the highest amplification rate. The direct relationship between cycle number and the log concentration of RNA molecules initially present in the RT-qPCR reaction was used to calibrate the crossing points of the amplification curves for the samples.

### Immunohistochemistry.

Frozen human duodenal tissue sections were obtained from OriGene Technologies (Rockville, MD). To determine the expression levels of α2,6-linked SA in the human small intestine and in the RVA-infected cells of the differentiated HIEs and BIEs, immunohistochemistry was performed as described previously (23, 29), with slight modifications. Briefly, either the human intestinal tissue sections fixed with 10% formalin or the HIE and BIE sections prepared as described above were washed three times with PBS. Blocking was performed using PBS containing 1% BSA for 30 min at room temperature; blocking buffer was also used as a diluent. Unless otherwise indicated, the following steps were performed at room temperature, with an incubation time of 1 h, and three PBS washes were performed between each step. Human intestinal tissue sections were stained for α2,6-SAs using biotinylated SNL (10 μg/ml), followed by DyLight 488-conjugated streptavidin. HIE and BIE sections were stained using an anti-RV VP6 protein MAb (1:100 dilution), followed by Alexa Fluor 488-conjugated donkey anti-mouse antibody (Invitrogen). Cell nuclei were stained with DAPI. To control the sialic acid dependence of the labeling, tissue sections were pretreated with 500 U of neuraminidase (New England Biolabs) for 4 h at 37°C. Positive control sections without NA treatment were preincubated with the manufacturer’s enzyme buffer only; the protocol described above was then performed. After a final wash with distilled water, coverslips were mounted on glass slides with Prolong gold antifade mountant (Thermo Fisher Scientific), and the slides were analyzed using a Nikon A1 RSI confocal microscope or LSM 510 confocal microscope.

### Statistical analysis.

Statistical analyses were performed using GraphPad Prism 5.03 (USA). A one-way analysis of variance was used to determine the statistical significance (*P* < 0.05).

### Ethical statements.

All animals were handled in strict accordance with good animal practices, as described in the NIH Guide for the Care and Use of Laboratory Animals (NIH Publication 85-23, 1985, revised 1996). The protocols for collecting bovine, porcine, chicken, rabbit, and mouse blood samples, for collecting bovine and porcine saliva samples, and for generating bovine enteroids were approved by the Committee on Ethics of Animal Experiments, Chonnam National University (CNU), with permit numbers CNU IACUC-YB-2016-13, CNU 2012-87, and CNU IACUC-YB-2016-65. The human blood and saliva samples were collected with written consent from the donors, while the human enteroids, kindly provided by M. Estes (Department of Molecular Virology and Microbiology, Baylor College of Medicine, Houston, TX), were handled in strict accordance with sampling of human subjects, as described in the Guidance for the Care and Use of Human Samples of CNU, which adheres to the WMA Declaration of Helsinki (Ethical Principles for Medical Research Involving Human Subjects). Protocols were approved by the Committee for Research Ethics Concerning Human Subjects, CNU, with permit numbers CNU IBR 1040198-130807-BR-002-01 and CNU IBR 1040198-170120-BR-002-01. The human duodenal frozen tissue sections obtained from OriGene Technologies were handled in strict accordance with sampling of human subjects, as described in the Guidance for the Care and Use of Human Samples of the Université de Nantes which adheres to the WMA Declaration of Helsinki (Ethical Principles for Medical Research Involving Human Subjects).

## References

[1] Liu L, Johnson HL, Cousens S, Perin J, Scott S, Lawn JE, Rudan I, Campbell H, Cibulskis R, Li M, Mathers C, Black RE 2012 Global, regional, and national causes of child mortality: an updated systematic analysis for 2010 with time trends since 2000. Lancet 379:2151–2161. doi:10.1016/S0140-6736(12)60560-1. 22579125

[2] Estes MK, Greenberg HB 2013 Rotaviruses, p 1917–1974. *In* Knipe DM, Howley PM (ed), Fields virology, 6th ed, vol 2 Lippincott/Williams & Wilkins, Philadelphia, PA.

[3] Martella V, Bányai K, Matthijnssens J, Buonavoglia C, Ciarlet M 2010 Zoonotic aspects of rotaviruses. Vet Microbiol 140:246–255. doi:10.1016/j.vetmic.2009.08.028. 19781872

[4] Walker CLF, Rudan I, Liu L, Nair H, Theodoratou E, Bhutta ZA, O’Brien KL, Campbell H, Black RE 2013 Global burden of childhood pneumonia and diarrhea. Lancet 381:1405–1416. doi:10.1016/S0140-6736(13)60222-6. 23582727 PMC7159282

[5] Arias CF, Silva-Ayala D, López S 2015 Rotavirus entry: a deep journey into the cell with several exits. J Virol 89:890–893. doi:10.1128/JVI.01787-14. 25378490 PMC4300671

[6] Estes MK, Graham DY, Mason BB 1981 Proteolytic enhancement of rotavirus infectivity: molecular mechanisms. J Virol 39:879–888. 6270356 10.1128/jvi.39.3.879-888.1981 PMC171321

[7] López S, Arias CF 2004 Multistep entry of rotavirus into cells: a Versaillesque dance. Trends Microbiol 12:271–278. doi:10.1016/j.tim.2004.04.003. 15165605

[8] Collins PJ, Martella V, Buonavoglia C, O’Shea H 2010 Identification of a G2-like porcine rotavirus bearing a novel VP4 type, P[32]. Vet Res 41:73. doi:10.1051/vetres/2010045. 20663474 PMC2939698

[9] Matthijnssens J, Ciarlet M, Heiman E, Arijs I, Delbeke T, McDonald SM, Palombo EA, Iturriza-Gómara M, Maes P, Patton JT, Rahman M, Van Ranst M 2008 Full genome-based classification of rotaviruses reveals a common origin between human Wa-like and porcine rotavirus strains and human DS-1-like and bovine rotavirus strains. J Virol 82:3204–3219. doi:10.1128/JVI.02257-07. 18216098 PMC2268446

[10] Matthijnssens J, Ciarlet M, McDonald SM, Attoui H, Banyai K, Brister JR, Buesa J, Esona MD, Estes MK, Gentsch JR, Iturriza-Gómora M, Johne R, Kirkwood CD, Martella V, Mertens PP, Nakagomi O, Parreño V, Rahman M, Ruggeri FM, Saif LJ, Santos N, Steyer A, Taniguchi K, Patton JT, Desselberger U, Van Ranst M 2011 Uniformity of rotavirus strain nomenclature proposed by the Rotavirus Classification Working Group (RCWG). Arch Virol 156:1397–1413. doi:10.1007/s00705-011-1006-z. 21597953 PMC3398998

[11] Rojas MA, Goncalves JLS, Dias HG, Manchego A, Santos N 2017 Identification of two novel rotavirus A genotypes, G35 and P[50], from Peruvian alpaca faeces. Infect Genet Evol 55:71–74. doi:10.1016/j.meegid.2017.08.019. 28866138 PMC7106126

[12] Trojnar E, Sachsenroder J, Twardziok S, Reetz J, Otto PH, Johne R 2013 Identification of an avian group A rotavirus containing a novel VP4 gene with a close relationship to those of mammalian rotaviruses. J Gen Virol 94:136–142. doi:10.1099/vir.0.047381-0. 23052396

[13] Yolken RH, Willoughby R, Wee SB, Miskuff R, Vonderfecht S 1987 Sialic acid glycoproteins inhibit *in vitro* and *in vivo* replication of rotaviruses. J Clin Invest 79:148–154. doi:10.1172/JCI112775. 3025257 PMC424010

[14] Ciarlet M, Estes MK 1999 Human and most animal rotavirus strains do not require the presence of sialic acid on the cell surface for efficient infectivity. J Gen Virol 80:943–948. doi:10.1099/0022-1317-80-4-943. 10211964

[15] Ciarlet M, Ludert JE, Iturriza-Gómara M, Liprandi F, Gray JJ, Desselberger U, Estes MK 2002 Initial interaction of rotavirus strains with *N*-acetylneuraminic (sialic) acid residues on the cell surface correlates with VP4 genotype, not species of origin. J Virol 76:4087–4095. doi:10.1128/JVI.76.8.4087-4095.2002. 11907248 PMC136071

[16] Guo CT, Nakagomi O, Mochizuki M, Ishida H, Kiso M, Ohta Y, Suzuki T, Miyamoto D, Hidari KI, Suzuki Y 1999 Ganglioside GM_1a_ on the cell surface is involved in the infection by human rotavirus KUN and MO strains. J Biochem 124:683–688. doi:10.1093/oxfordjournals.jbchem.a022503. 10502675

[17] Haselhorst T, Fleming FE, Dyason JC, Hartnell RD, Yu X, Holloway G, Santegoets K, Kiefel MJ, Blanchard H, Coulson BS, von Itzstein M 2009 Sialic acid dependence in rotavirus host cell invasion. Nat Chem Biol 5:91–93. doi:10.1038/nchembio.134. 19109595

[18] Böhm R, Fleming FE, Maggioni A, Dang VT, Holloway G, Coulson BS, von Itzstein M, Haselhorst T 2015 Revisiting the role of histo-blood group antigens in rotavirus host-cell invasion. Nat Commun 6:5907. doi:10.1038/ncomms6907. 25556995

[19] Hu L, Crawford SE, Czako R, Cortes-Penfield NW, Smith DF, Le Pendu J, Estes MK, Prasad BV 2012 Cell attachment protein VP8* of a human rotavirus specifically interacts with A-type histo-blood group antigen. Nature 485:256–259. doi:10.1038/nature10996. 22504179 PMC3350622

[20] Huang P, Xia M, Tan M, Zhong W, Wei C, Wang L, Morrow A, Jiang X 2012 Spike protein VP8* of human rotavirus recognizes histo-blood group antigens in a type-specific manner. J Virol 86:4833–4843. doi:10.1128/JVI.05507-11. 22345472 PMC3347384

[21] Liu Y, Huang P, Tan M, Liu Y, Biesiada J, Meller J, Castello AA, Jiang B, Jiang X 2012 Rotavirus VP8*, phylogeny, host range, and interaction with histo-blood group antigens. J Virol 86:9899–9910. doi:10.1128/JVI.00979-12. 22761376 PMC3446626

[22] Tan M, Jiang X 2014 Histo-blood group antigens: a common niche for norovirus and rotavirus. Expert Rev Mol Med 16:e5. doi:10.1017/erm.2014.2. 24606759 PMC12406300

[23] Barbé L, Le Moullac-Vaidye B, Echasserieau K, Bernardeau K, Carton T, Bovin N, Nordgren J, Svensson L, Ruvoën-Clouet N, Le Pendu J 2018 Histo-blood group antigen-binding specificities of human rotaviruses are associated with gastroenteritis but not with *in vitro* infection. Sci Rep 8:12961. doi:10.1038/s41598-018-31005-4. 30154494 PMC6113245

[24] Ramani S, Cortes-Penfield NW, Hu L, Crawford SE, Czako R, Smith DF, Kang G, Ramig RF, Le Pendu J, Prasad BV, Estes MK 2013 The VP8* domain of neonatal rotavirus strain G10P[11] binds to type II precursor glycans. J Virol 87:7255–7264. doi:10.1128/JVI.03518-12. 23616650 PMC3700318

[25] Ciarlet M, Schödel F 2009 Development of a rotavirus vaccine: clinical safety, immunogenicity, and efficacy of the pentavalent rotavirus vaccine, RotaTeq. Vaccine 27:G72–G81. doi:10.1016/j.vaccine.2009.09.107. 20006144

[26] Isa P, Arias CF, López S 2006 Role of sialic acids in rotavirus infection. Glycoconj J 23:27–37. doi:10.1007/s10719-006-5435-y. 16575520 PMC7087688

[27] Hu L, Sankaran B, Laucirica DR, Patil K, Salmen W, Ferreon ACM, Tsoi PS, Lasanajak Y, Smith DF, Ramani S, Atmar RL, Estes MK, Ferreon JC, Prasad B 2018 Glycan recognition in globally dominant human rotaviruses. Nat Commun 9:2631. doi:10.1038/s41467-018-05098-4. 29980685 PMC6035239

[28] Macher BA, Galili U 2008 The Galα1,3Galβ1,4GlcNAc-R (α-Gal) epitope: a carbohydrate of unique evolution and clinical relevance. Biochim Biophys Acta 1780:75–88. doi:10.1016/j.bbagen.2007.11.003. 18047841 PMC2271034

[29] Zakhour M, Ruvoen-Clouët N, Charpilienne A, Langpap B, Poncet D, Peters T, Bovin N, Le Pendu J 2009 The αGal epitope of the histo-blood group antigen family is a ligand for bovine norovirus Newbury2 expected to prevent cross-species transmission. PLoS Pathog 5:e1000504. doi:10.1371/journal.ppat.1000504. 19578439 PMC2699481

[30] Varki A 2001 Loss of *N*-glycolylneuraminic acid in humans: mechanisms, consequences, and implications for hominid evolution. Am J Phys Anthropol 33:54–69. doi:10.1002/ajpa.10018. 11786991 PMC7159735

[31] Saxena K, Blutt SE, Ettayebi K, Zeng XL, Broughman JR, Crawford SE, Karandikar UC, Sastri NP, Conner ME, Opekun AR, Graham DY, Qureshi W, Sherman V, Foulke-Abel J, In J, Kovbasnjuk O, Zachos NC, Donowitz M, Estes MK 2016 Human intestinal enteroids: a new model to study human rotavirus infection, host restriction, and pathophysiology. J Virol 90:43–56. doi:10.1128/JVI.01930-15. 26446608 PMC4702582

[32] Ettayebi K, Crawford SE, Murakami K, Broughman JR, Karandikar U, Tenge VR, Neill FH, Blutt SE, Zeng X-L, Qu L, Kou B, Opekun AR, Burrin D, Graham DY, Ramani S, Atmar RL, Estes MK 2016 Replication of human noroviruses in stem cell-derived human enteroids. Science 353:1387–1393. doi:10.1126/science.aaf5211. 27562956 PMC5305121

[33] Heise MT, Virgin HW 2013 Pathogenesis of viral infection, p 254–285. *In* Knipe DM, Howley PM (ed), Fields virology, 6th ed, vol 1 Lippincott/Williams & Wilkins, Philadelphia, PA.

[34] Olofsson S, Bergström T 2005 Glycoconjugateglycans as viral receptors. Ann Med 37:154–172. doi:10.1080/07853890510007340. 16019714

[35] Cook N, Bridger J, Kendall K, Gomar M, El AL, Gray J 2004 The zoonotic potential of rotavirus. J Infect 48:289–302. doi:10.1016/j.jinf.2004.01.018. 15066329

[36] Dhama K, Chauhan RS, Mahendran M, Malik S 2009 Rotavirus diarrhea in bovines and other domestic animals. Vet Res Commun 33:1–23. doi:10.1007/s11259-008-9070-x. PMC7088678 18622713

[37] Matthijnssens J, Rahman M, Martella V, Xuelei Y, De Vos S, De Leener K, Ciarlet M, Buonavoglia C, Van Ranst M 2006 Full genomic analysis of human rotavirus strain B4106 and lapine rotavirus strain 30/96 provides evidence for interspecies transmission. J Virol 80:3801–3810. doi:10.1128/JVI.80.8.3801-3810.2006. 16571797 PMC1440464

[38] Santos N, Hoshino Y 2005 Global distribution of rotavirus serotypes/genotypes and its implication for the development and implementation of an effective rotavirus vaccine. Rev Med Virol 15:29–56. doi:10.1002/rmv.448. 15484186

[39] Clark HF, Offit PA, Ellis RW, Eiden JJ, Krah D, Shaw AR, Pichichero M, Treanor JJ, Borian FE, Bell LM, Plotkin SA 1996 The development of multivalent bovine rotavirus (strain WC3) reassortant vaccine for infants. J Infect Dis 174:S73–S80. doi:10.1093/infdis/174.Supplement_1.S73. 8752294

[40] Vesikari T, Matson DO, Dennehy P, Van Damme P, Santosham M, Rodriguez Z, Dallas MJ, Heyse JF, Goveia MG, Black SB, Shinefield HR, Christie CDC, Ylitalo S, Itzler RF, Coia ML, Onorato MT, Adeyi BA, Marshall GS, Gothefors L, Campens D, Karvonen A, Watt JP, O’Brien KL, DiNubile MJ, Clark HF, Boslego JW, Offit PA, Heaton PM, Rotavirus Efficacy and Safety Trial (UREST) Study Team. 2006 Safety and efficacy of a pentavalent human-bovine (WC3) reassortant rotavirus vaccine. N Engl J Med 354:23–33. doi:10.1056/NEJMoa052664. 16394299

[41] Kim DS, Hosmillo M, Alfajaro MM, Kim JY, Park JG, Son KY, Ryu EH, Sorgeloos F, Kwon HJ, Park SJ, Lee WS, Cho D, Kwon J, Choi JS, Kang MI, Goodfellow I, Cho KO 2014 Both α2,3- and 2,6-linked sialic acids on O-linked glycoproteins act as functional receptors for porcine sapovirus. PLoS Pathog 10:e1004172. doi:10.1371/journal.ppat.1004172. 24901849 PMC4047124

[42] Kim D-S, Son K-Y, Koo K-M, Kim J-Y, Alfajaro MM, Park J-G, Hosmillo M, Soliman M, Baek Y-B, Cho E-H, Lee J-H, Kang M-I, Goodfellow I, Cho K-O 2016 Porcine sapelovirus uses α2,3-linked sialic acid on GD1a ganglioside as a receptor. J Virol 90:4067–4077. doi:10.1128/JVI.02449-15. 26865725 PMC4810533

[43] Stuart AD, Brown TD 2007 Alpha2,6-linked sialic acid acts as a receptor for feline calicivirus. J Gen Virol 88:177–186. doi:10.1099/vir.0.82158-0. 17170450

[44] Méndez E, Arias CF, López S 1996 Interactions between the two surface proteins of rotavirus may alter the receptor-binding specificity of the virus. J Virol 70:1218–1222. 8551583 10.1128/jvi.70.2.1218-1222.1996 PMC189931

[45] Farkas T, Cross RW, Hargitt E III, Lerche NW, Morrow AL, Sestak K 2010 Genetic diversity and histo-blood group antigen interactions of rhesus enteric caliciviruses. J Virol 84:8617–8625. doi:10.1128/JVI.00630-10. 20554772 PMC2919043

[46] Nyström K, Le Gall-Reculé G, Grassi P, Abrantes J, Ruvoën-Clouet N, Le Moullac-Vaidye B, Lopes AM, Esteves PJ, Strive T, MArchandeau S, Dell A, Haslam SM, Le Pendu J 2011 Histo-blood group antigens act as attachment factors of rabbit hemorrhagic disease virus infection in a virus strain-dependent manner. PLoS Pathog 7:e1002188. doi:10.1371/journal.ppat.1002188. 21901093 PMC3161982

[47] Tan M, Jiang X 2010 Norovirus gastroenteritis, carbohydrate receptors, and animal models. PLoS Pathog 6:e1000983. doi:10.1371/journal.ppat.1000983. 20865168 PMC2928792

[48] Taube S, Perry JW, Yetming K, Patel SP, Auble H, Shu L, Nawar HF, Lee CH, Connell TD, Shayman JA, Wobus CE 2009 Ganglioside-linked terminal sialic acid moieties on murine macrophages function as attachment receptors for murine noroviruses (MNV). J Virol 83:4092–4101. doi:10.1128/JVI.02245-08. 19244326 PMC2668497

[49] Zhang D, Huang P, Zou L, Lowary TL, Tan M, Jiang X 2015 Tulane virus recognizes the A type 3 and B histo-blood group antigens. J Virol 89:1419–1427. doi:10.1128/JVI.02595-14. 25392226 PMC4300669

[50] Mochizuki M, Nakagomi O 1995 Haemagglutination by rotaviruses in relation to VP4 genotypes. Res Virol 146:371–374. doi:10.1016/0923-2516(96)80600-5. 8578011

[51] Teneberg S, Willemsen PT, de Graaf FK, Stenhagen G, Pimlott W, Jovall PA, Angström J, Karlsson KA 1994 Characterization of gangliosides of epithelial cells of calf small intestine, with special reference to receptor-active sequences for enteropathogenic *Escherichia coli* K99. J Biochem 116:560–574. doi:10.1093/oxfordjournals.jbchem.a124562. 7531686

[52] Neu U, Bauer J, Stehle T 2011 Viruses and sialic acids: rules of engagement. Curr Opin Struct Biol 21:610–618. doi:10.1016/j.sbi.2011.08.009. 21917445 PMC3189341

[53] Stehle T, Khan ZM 2014 Rules and exceptions: sialic acid variants and their role in determining viral tropism. J Virol 88:7696–7699. doi:10.1128/JVI.03683-13. 24807712 PMC4097780

[54] Breimer ME, Hansson GC, Karlsson KA, Larson G, Leffler H 2012 Glycosphingolipid composition of epithelial cells isolated along the villus axis of small intestine of a single human individual. Glycobiology 22:1721–1730. doi:10.1093/glycob/cws115. 22833314

[55] Kaneko Y, Yamamoto H, Colley KJ, Moskal JR 1995 Expression of Galβ1,4GlcNAc α2,6-sialyltransferase and α2,6-linked sialoglycoconjugates in normal human and rat tissues. J Histochem Cytochem 43:945–954. doi:10.1177/43.9.7642967. 7642967

[56] Robbe C, Capon C, Coddeville B, Michalski JC 2004 Structural diversity and specific distribution of O-glycans in normal human mucins along the intestinal tract. Biochem J 384:307–316. doi:10.1042/BJ20040605. 15361072 PMC1134114

[57] Yao L, Korteweg C, Hsueh W, Gu J 2008 Avian influenza receptor expression in H5N1-infected and noninfected human tissues. FASEB J 22:733–740. doi:10.1096/fj.06-7880com. 17925493

[58] Armah GE, Cortese MM, Dennis FE, Yu Y, Morrow AL, McNeal MM, Lewis KDC, Awuni DA, Armachie J, Parashar UD 2019 Rotavirus vaccine take in infants is associated with secretor status. J Infect Dis 219:746–749. doi:10.1093/infdis/jiy573. 30357332

[59] Bucardo F, Nordgren J, Reyes Y, Gonzalez F, Sharma S, Svensson L 2018 The Lewis A phenotype is a restriction factor for rotate and rotarix vaccine-take in Nicaraguan children. Sci Rep 8:1509. doi:10.1038/s41598-018-19718-y. 29367698 PMC5784145

[60] Kazi AM, Cortese MM, Yu Y, Lopman B, Morrow AL, Fleming JA, McNeal MM, Steele AD, Parashar UD, Zaidi AKM, Ali A 2017 Secretor and salivary ABO blood group antigen status predict rotavirus vaccine take in infants. J Infect Dis 215:786–789. doi:10.1093/infdis/jix028. 28329092

[61] Zarate S, Espinosa R, Romero P, Méndez E, Arias CF, Lopez S 2000 The VP5 domain of VP4 can mediate attachment of rotavirus to cells. J Virol 74:594–599. 10.1128/jvi.74.2.593-599.2000 PMC111578 10623720

[62] Desselberger U 2017 Reverse genetics of rotavirus. Proc Natl Acad Sci U S A 114:2106–2108. doi:10.1073/pnas.1700738114. 28183796 PMC5338543

[63] Kanai Y, Komoto S, Kawagishi T, Nouda R, Nagasawa N, Onishi M, Matsuura Y, Taniguchi K, Kobayashi T 2017 Entirely plasmid-based reverse genetics system for rotaviruses. Proc Natl Acad Sci U S A 114:2349–2354. doi:10.1073/pnas.1618424114. 28137864 PMC5338561

[64] Komoto S, Fukuda S, Ide T, Ito N, Sugiyama M, Yoshikawa T, Murata T, Taniguchi K 2018 Generation of recombinant rotaviruses expressing fluorescent proteins by using an optimized reverse genetics system. J Virol 92:e00588-18. doi:10.1128/JVI.00588-18. 29669834 PMC6002737

[65] Kim HJ, Park JG, Alfajaro MM, Kim DS, Hosmillo M, Son KY, Lee JH, Bae YC, Park SI, Kang MI, Cho KO 2012 Pathogenicity characterization of a bovine triple reassortant rotavirus in calves and piglets. Vet Microbiol 159:11–22. doi:10.1016/j.vetmic.2012.03.017. 22465801

[66] Nokhbeh MR, Hazra S, Alexander DA, Khan A, McAllister M, Suuronen EJ, Griffith M, Dimock K 2005 Enterovirus 70 binds to different glycoconjugates containing α2,3-linked sialic acid on different cell lines. J Virol 79:7087–7094. doi:10.1128/JVI.79.11.7087-7094.2005. 15890948 PMC1112099

[67] Tan M, Meller J, Jiang X 2006 C-terminal arginine cluster is essential for receptor binding of norovirus capsid protein. J Virol 80:7322–7331. doi:10.1128/JVI.00233-06. 16840313 PMC1563700

[68] Park JG, Kim HJ, Matthijnssens J, Alfajaro MM, Kim DS, Son KY, Kwon HJ, Hosmillo M, Ryu EH, Kim JY, Cena RB, Lee JH, Kang MI, Park SI, Cho KO 2013 Different virulence of porcine and porcine-like bovine rotavirus strains with genetically nearly identical genomes in piglets and calves. Vet Res 44:88. doi:10.1186/1297-9716-44-88. 24083947 PMC3851489

[69] Pieper U, Webb BM, Barkan DT, Schneidman-Duhovny D, Schlessinger A, Braberg H, Yang Z, Meng EC, Pettersen EF, Huang CC, Datta RS, Sampathkumar P, Madhusudhan MS, Sjölander K, Ferrin TE, Burley SK, Sali A 2011 ModBase, a database of annotated comparative protein structure models, and associated resources. Nucleic Acids Res 39:D465–D474. doi:10.1093/nar/gkq1091. 21097780 PMC3013688

[70] Cho EH, Soliman M, Alfajaro MM, Kim JY, Seo JY, Park JG, Kim DS, Baek YB, Kang MI, Park SI, Le Pendu J, Cho KO 2018 Bovine nebovirus interacts with a wide spectrum of histo-blood group antigens. J Virol 92:e02160-17. doi:10.1128/JVI.02160-17. 29467317 PMC5899197

[71] Kim DS, Kang MI, Son KY, Bak GY, Park JG, Hosmillo M, Seo JY, Kim JY, Alfajaro MM, Soliman M, Baek YB, Cho EH, Lee JH, Kwon J, Choi JS, Goodfellow I, Cho KO 2016 Pathogenesis of Korean sapelovirus A in piglets and chicks. J Gen Virol 97:2566–2574. doi:10.1099/jgv.0.000571. 27487773 PMC5078829

[72] Huang P, Farkas T, Marionneau S, Zhong W, Ruvoen-Clouët N, Morrow AL, Altaye M, Pickering LK, Newburg DS, Le Pendu J, Jiang X 2003 Noroviruses bind to human ABO, Lewis, and secretor histo-blood group antigens: identification of 4 distinct strain-specific patterns. J Infect Dis 188:19–31. doi:10.1086/375742. 12825167

[73] Huang P, Farkas T, Zhong W, Tan M, Thornton S, Morrow AL, Jiang X 2005 Norovirus and histo-blood group antigens: demonstration of a wide spectrum of strain specificities and classification of two major binding groups among multiple binding patterns. J Virol 79:6714–6722. doi:10.1128/JVI.79.11.6714-6722.2005. 15890909 PMC1112114

[74] Soliman M, Seo JY, Kim DS, Kim JY, Park JG, Alfajaro MM, Baek YB, Cho EH, Kwon J, Choi JS, Kang MI, Park SI, Cho KO 2018 Activation of PI3K, Akt, and ERK during early rotavirus infection leads to V-ATPase-dependent endosomal acidification required for uncoating. PLoS Pathog 14:e1006820. doi:10.1371/journal.ppat.1006820. 29352319 PMC5792019

